# Butterflies in the gut: the interplay between intestinal microbiota and stress

**DOI:** 10.1186/s12929-023-00984-6

**Published:** 2023-11-28

**Authors:** Tzu-Ting Lai, Chia-Wei Liou, Yu-Hsuan Tsai, Yuan-Yuan Lin, Wei-Li Wu

**Affiliations:** 1 Department of Physiology, College of Medicine, National Cheng Kung University, 1 University Rd., Tainan, 70101 Taiwan; 2 Institute of Basic Medical Sciences, College of Medicine, National Cheng Kung University, 1 University Rd., Tainan, 70101 Taiwan

**Keywords:** Gut-brain axis, Microbiota, Microbiome, Stress, Corticosterone, Intestinal steroidogenesis, Neural circuits, Autonomic nervous system, Probiotic, Prebiotic

## Abstract

Psychological stress is a global issue that affects at least one-third of the population worldwide and increases the risk of numerous psychiatric disorders. Accumulating evidence suggests that the gut and its inhabiting microbes may regulate stress and stress-associated behavioral abnormalities. Hence, the objective of this review is to explore the causal relationships between the gut microbiota, stress, and behavior. Dysbiosis of the microbiome after stress exposure indicated microbial adaption to stressors. Strikingly, the hyperactivated stress signaling found in microbiota-deficient rodents can be normalized by microbiota-based treatments, suggesting that gut microbiota can actively modify the stress response. Microbiota can regulate stress response via intestinal glucocorticoids or autonomic nervous system. Several studies suggest that gut bacteria are involved in the direct modulation of steroid synthesis and metabolism. This review provides recent discoveries on the pathways by which gut microbes affect stress signaling and brain circuits and ultimately impact the host’s complex behavior.

## Introduction

The etymology for the phrase to have “butterflies in the stomach” first appeared in the book “*The House of Prayer*” written by Florence Converse in 1908. This phrase has been widely used as an idiom for over a hundred years, and it describes an unsettling feeling when one is facing a stressful or thrilling event. It is particularly fascinating that people describe this feeling as something that originates in the gut, and not elsewhere. Scientists have been chasing these “butterflies” and their origins for over two decades now, and they realized that this idiom may be associated with a feeling and sensation that is influenced by the commensal microbes in the gastrointestinal (GI) tract. Scientists have made amazing discoveries about understanding the importance of commensal gut microbes in host physiology and pathophysiology.

The flopping butterfly is not only a metaphor for the fluttery feeling in our body, but it is also a term that describes the initial action in a series of chain reactions for a colossal event. Commensal microbes in the gut exert various effects on host behavior through the “gut-brain axis.” The “gut-brain axis” is the distal connection between the GI system and the central nervous system [1]; it is composed of complex signal transduction pathways across the two body systems [2]. Gut bacteria and their metabolites exert their “butterfly effect,” which propagates signals to the brain, ultimately altering the host’s behavior. The hypothalamic–pituitary–adrenal (HPA) axis, the canonical pathway for stress regulation, is one of the most promising routes that connects the commensal gut microbes, GI tract, brain, and behavior to each other [2]; this also reflects the fluttery feeling in the gut. Moreover, stress signaling can be transmitted to the brain via the vagus nerve and afferent/efferent neuron connections.

Stressed, nervous, tense, worried, and anxious are commonly felt in the presence of threats. Recent findings suggest that the stress response and gut microbes reciprocally influence numerous behavioral outcomes in the host. To understand the role of commensal gut microbes in stress regulation and response, the use of gnotobiotic animals, 16S rRNA sequencing, metagenomic sequencing, fecal microbiota transplantation, antibiotic treatment, and probiotics are employed to unravel intertwined host-microbe interactions [2]. This review focuses on rodents as a model organism to explore the causal relationships between the gut microbiota, stress, and behavior. Some clinical observations have also been incorporated to support this review.

### Brain response to stress exposure

Stress sensing, integration, and coping are vital functions of the brain when confronted with an aversive stimulus [3, 4]. Stress-related information is integrated into the sensory cortex, which then sends signals to the limbic system, hypothalamus, and brainstem to activate the HPA axis and sympathetic and parasympathetic nerves [3, 5]. The sympathetic and parasympathetic nerves propagate the stress response to evoke rapid adaption in various systems in the body [3]. The brain regions that detect stress signals from the external environment overlap with the brain regions that participate in emotion, which coherently orchestrates the stress responses in animals [3, 6].

**Fig. 1:**
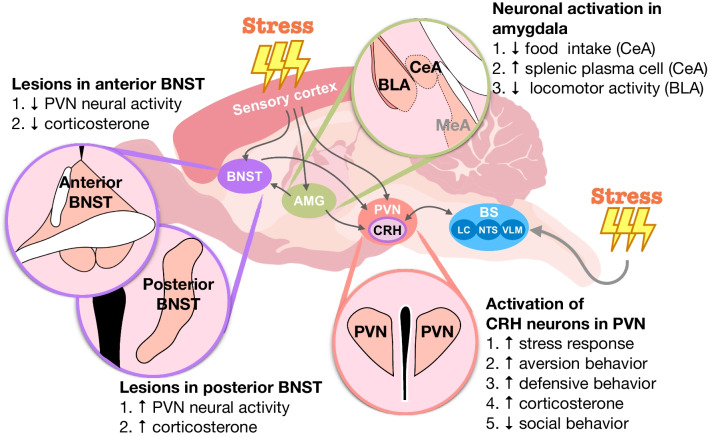
The orchestra of the paraventricular nucleus of the hypothalamus (PVN) with other brain regions in response to the stress exposure. CRH: corticotropin-releasing hormone; BNST: the bed nucleus of the stria terminalis; AMG: amygdala; CeA: central nucleus of the amygdala; BLA: basolateral amygdala; MeA: medial amygdala; BS: brainstem; LC: locus coeruleus; NTS: nucleus tractus solitarius; VLM: ventrolateral medulla

Among the brain regions that are involved in regulating the stress response, the paraventricular nucleus of the hypothalamus (PVN) plays a central role in integrating signals from the environmental stimuli and further triggering downstream neural transmission [3, 7]. The PVN receives neural innervation from the limbic system and brainstem to mediate the HPA axis and integrate the response after exposure to stress [3, 7]. Various types of neurons are located in the PVN. Primarily, the corticotrophin-releasing hormone (CRH) neurons in the PVN and other associated brain regions respond to different forms of stress [8, 9]. In Fig. 1, we summarize the findings about the interplay of CRH neurons in the PVN and cells at the bed nucleus of the stria terminalis (BNST) and amygdala in response to stress. These brain regions are crucial for determining the levels of circulating corticosterone and animal behavioral outputs.

As a central hub for the stress response, PVN CRH neurons can be triggered by stressful stimuli and subsequently, evoke several intrinsic behavioral responses [10–12]. This section focuses on the cause-and-effect relationships between the PVN CRH neurons and stress coping behaviors. Daviu et al. showed that an increase in CRH neuron activity in the PVN can be detected during a looming-shadow task, a method that mimics predator threat from the sky, where the majority of mice displayed escape behavior with little freezing response to a looming shadow. Silencing the PVN CRH neurons decreased the escape behavior but increased the freezing response. Moreover, PVN CRH neurons anticipate an imminent threat and encode stress controllability [11]. Similarly, another study showed that CRH neurons in the PVN responded to aversive stimuli, such as forced swimming, tail restraint, overhead objects, looming, and even intraperitoneal injection [13]. In one study done by Huang et al., mice susceptible to visceral pain after maternal separation exhibited an increased number of c-Fos-positive CRH neurons in PVN compared to resilient mice [14]. Electrophysiological recordings also revealed higher spontaneous firing frequency of CRH neurons in the PVN and increased evoked firing rates in response to step current injections in mice susceptible to visceral pain after maternal separation [14]. Moreover, susceptible mice displayed elevated gene expression and protein levels of CRH in the PVN, along with higher concentrations of CRH, ACTH, and corticosterone in the serum [14]. Additionally, exposure to water avoidance stress (WAS), an acute stress paradigm, induced an increase in c-Fos-positive cells in the PVN [15, 16]. The stress response to WAS was ameliorated by intracisternal injection of a CRH receptor antagonist [15, 16].

Mice displayed altered home-cage behavior, including self-grooming, rearing, walking, digging, and chewing, immediately after the footshock. Fuzesi et al. demonstrated that optogenetically activating the CRH neurons in the PVN increased self-grooming, shifting other home-cage behaviors similar to mice experiencing foot shock. The increased self-grooming behavior by the optogenetic stimulation of PVN CRH neurons can be attenuated by increasing the presumptive threat level of the context (testing environment) [12]. Furthermore, Sterley et al. found that the transmission of stress signaling from a stressed subject to a naive partner required the activation of PVN CRH neurons in both subjects and partners to drive stress signal transmission [17]. Similarly, Wu et al. showed that the chemogenetic activation of CRH neurons in the PVN during a social interaction test abolished social behavior and increased digging behavior in mice. Moreover, corticosterone levels increased after social interaction when PVN CRH neurons were activated [18]. In contrast, not all stress responses are associated with the PVN CRH neurons. Zhao et al. found that optogenetic activation of excitatory projections from the PVN to the ventrolateral medulla (VLM), but not CRH neurons in the PVN, could recapitulate stress-induced hyperglycemia in mice without stress exposure [19]. Nonetheless, these studies demonstrate that PVN CRH neurons are essential for controlling stress responses and behaviors when exposed to imminent threats (Fig. 1).

The BNST serves as a relay station that connects the various brain regions involved in emotion [20]. Amygdala-BNST and BNST-PVN circuits participate in stress response regulation [21, 22]. Previous studies have shown that the BNST is composed of several subregions and sends various projections to the PVN [20–23]. Duan et al. demonstrated that optogenetic activation of the basolateral amygdala (BLA) in the BNST circuit prevented anxiety-like behaviors in mice that received social defeat stress [24]. The anterior part of the BNST lesions inhibits the activation of the PVN and HPA axis after stressor exposure [25, 26]. Conversely, Choi et al. showed that lesions in the posterior part of the BNST increased corticosterone levels and the number of c-Fos-positive cells in the PVN after acute restraint stress [27]. Stress exposure also affects neural activity in the BNST. Predator stress, elevated plus maze, and restraint stress enhance the neural activity of CRH neurons in the BNST [28, 29]. However, Wu et al. showed that the inhibition of CRH neurons in the BNST could not rescue stress-induced social deficits [18], which suggests that the BNST might be affected by stress exposure but does not directly regulate the stress response.

The amygdala is a critical structure that is associated with emotional processing and physiological responses to stress [30, 31]. Various subregions of the amygdala participate in distinct mechanisms to modulate different types of stressor exposure [30]. Acute psychological stress increases the number of c-Fos-positive cells in the medial amygdala (MeA) [32] and enhances inhibitory neuron activity in the central nucleus of the amygdala (CeA) [33]. However, limited direct connections between the amygdala and PVN can mediate the stress response [3, 30]. The stress-induced immune dysregulation is associated with distinct neuronal populations in the CeA. Zhang et al. identified a circuit between the CeA/PVN and splenic nerve in the regulation of stress-associated immunity [34]. Artificial activation of CRH neurons in the CeA and PVN increases splenic plasma cell formation. Placing the mouse on the elevated platform not only increased the CRH neuronal activity, but also promoted splenic plasma cell formation, suggesting that the CeA and PVN participated in stress-induced immune response [34]. Furthermore, Xu et al. showed that CeA lesions prevented the release of CRH and adrenocorticotropic hormone (ACTH) after systemic interleukin (IL)-1 injection [35]. Other studies have also shown that peripheral injection of lipopolysaccharide (LPS) increased neural activity in the CeA to decrease food intake [36] and in the BLA to increase anxiety- and depressive-like behavior [37]. CRH neurons in other brain regions have been shown to play a role in stress response. Predator stimuli promote rapid arousal from rapid eye movement sleep in mice. A recent study by Tseng et al. showed that CRH neurons in the medial subthalamic nucleus (mSTN) were activated during rapid eye movement sleep by predator odor exposure in response to external stimuli [38]. The inhibition of CRH neurons in the mSTN increased the latency of freezing and looming behavior when the mice were exposed to predator odor and decreased the duration of the rapid eye movement-sleep response to adapt to the predator threats [38].

Overall, stress exposure triggered the orchestra of PVN CRH neurons with other brain regions in response to various stimuli (Fig. 1). PVN CRH neurons appear to be central hubs that connect other brain areas to initiate stress responses and coping mechanisms. Understanding the central pathway of the stress response is important in discovering the signaling pathway that is modulated by gut microbes.

### Stress exposure alters gut microbiome

Stress-coping mechanisms and adaptation are critical for survival. Animals cope with stress in many ways, including changes in their physiology and behavior. Interestingly, scientists have found that stress exposure affects the gut microbiome using rodent models (Table 1).

**Table 1:** Adaptation of commensal microbiome and behavior under acute and chronic stress conditions

Stress model	Stress period	Strain	Age	Sex	Vendor	Food	Sample	Trend	Rank	Identified bacteria	Phylum	Fold change	Behavior test	Behavior test outcome	Reference (PMID)
UCMS	8w	C57BL/6J	16w	Male	Janvier laboratory	N/A	Feces	Decrease	f	*Lactobacillaceae*	*Firmicutes*	N/A	Novelty suppressed feeding test	Increased time of latency to eat	33311466
													Splash test	Increased time of latency to groom	
													Tail suspension test	Increased time of immobility	
													Forced swimming test	Increased time of immobility	
													Light/dark box test	No change	
UCMS	5w	C57BL/6J	14w	Male	Jackson laboratory	N/A	Feces	Increase	g	*Cyanobacterium*	*Cyanobacteria*	3803.0271	Forced swimming test	Decreased time of escape behavior	28266612
									g	*Allobaculum*	*Firmicutes*	515.0243	Open-field test	No change	
									g	*Bifidobacterium*	*Actinobacteria*	70.4967			
									g	*Anaerofustis*	*Firmicutes*	8.4009			
									g	*Clostridium*	*Firmicutes*	7.2681			
									g	*Ruminococcus*	*Firmicutes*	6.1411			
									f	*Lachnospiraceae*	*Firmicutes*	2.9609			
									f	*Erysipelotrichaceae*	*Firmicutes*	2.2803			
								Decrease	g	*Lactobacillus*	*Firmicutes*	7.9232			
									g	*Anaeroplasma*	*Tenericutes*	7.1972			
									f	*Turicibacteraceae*	*Firmicutes*	5.6748			
									g	*Coprococcus*	*Firmicutes*	3.8667			
									c	Mollicutes	Tenericutes	3.5037			
									f	*Peptococcaceae*	*Firmicutes*	2.9847			
									g	*Eubacterium*	*Firmicutes*	2.7191			
									o	Clostridiales	Firmicutes	1.8332			
									f	*Catabacteriaceae*	*Firmicutes*	1.8304			
UCMS	8w	C57BL/6J	16w	Male	Janvier laboratory	N/A	Feces	Decrease	p	*Firmicutes*	*Firmicutes*	N/A	Tail suspension test	Increased time of immobility	32187541
									p	*Tenericutes*	*Tenericutes*	N/A	Forced swimming test	Increased time of immobility	
									p	*Saccharibacteria*	*Saccharibacteria*	N/A			
CSDS	5m/d, 1w	C57BL/6J	9w	Male	Jackson laboratory	Prolab® RMH 3500	Feces	Increase	p	*Mucispirillum*	*Mucispirillum*	326.3333	Open-field test	Decreased the travel distance	30824791
						LabDiet, St. Louis, MO			g	*Candidatus Arthromitus*	*Firmicutes*	181.0000	Open-field test	Decreased time spent in center zone	
									g	*Bilophila*	*Proteobacteria*	24.5000	Sucrose preference test	Decreased sucrose preference	
									g	*Helicobacter*	*Proteobacteria*	11.8182	Forced swimming test	Increased time of immobility	
									g	*Flexispira*	*Proteobacteria*	9.7307			
									g	*Odoribacter*	*Bacteroidetes*	7.9181			
									g	*Dehalobacterium*	*Firmicutes*	1.8105			
									g	*Coprococcus*	*Firmicutes*	1.7233			
									g	*Ruminococcus*	*Firmicutes*	1.6582			
									g	*Oscillospira*	*Firmicutes*	1.6153			
								Decrease	g	*Turicibacter*	*Firmicutes*	26.3000			
									g	*Paraprevotella*	*Bacteroidetes*	16.0202			
									g	*Allobaculum*	*Firmicutes*	9.0435			
									g	*Bifidobacterium*	*Actinobacteria*	8.6522			
									g	*Akkermansia*	*Verrucomicrobiota*	4.0375			
									f	*Mogibacteriaceae*	*Firmicutes*	2.6246			
									f	*Nitrosomonadaceae*	*Proteobacteria*	2.5000			
									f	*Coriobacteriaceae*	*Actinobacteria*	2.3158			
									g	*Anaerostipes*	*Firmicutes*	2.1546			
									g	*Dorea*	*Firmicutes*	2.1289			
CSDS	5m/d, 10d	C57BL/6J	8–9w	Male	National Laboratory Animal Center	N/A	Feces	Increase	f	*Bacteroidales S24–7*	*Bacteroidetes*	N/A	Social interaction test	Decreased the social index	34327733
					NLAC, Taipei, Taiwan				f	*Porphyromonadaceae*	*Bacteroidetes*	N/A	Open-field test	Decreased the travel distance	
									g	*Bacteroides*	*Bacteroidetes*	N/A	Open-field test	Decreased time spent in center zone	
								Decrease	g	*Enterorhabdus*	*Actinobacteria*	N/A	Sucrose preference test	Decreased sucrose preference	
									f	*Unclassified Bacteroidales S24-7*	*Bacteroidetes*	N/A			
									f	*Ruminococcaceae*	*Firmicutes*	N/A			
CSDS	5m/d, 10d	C57BL/6J	12–13w	Male	Charles River Japan	CE-2, CLEA Japan	Feces	Increase	p	*Bacteroidetes*	*Bacteroidetes*	N/A	Social interaction test	Decreased the social time	33972646
						Tokyo, Japan			p	*Actinobacteria*	*Actinobacteria*	N/A		Decreased distance traveled	
									p	*Proteobacteria*	*Proteobacteria*	N/A			
								Decrease	p	*Firmicutes*	*Firmicutes*	N/A			
SOC	10 mice/cage, 19w	C57BL/6J	23w	Male	Jackson laboratory	N/A	Feces	Increase	g	*Allobaculum*	*Firmicutes*	LDA score	Open-field test	No difference	34856844
									f	*Verrucomicrobiaceae*	*Verrucomicrobia*	(high to low)	Elevated plus maze	Increased of the speed	
									c	Verrucomicrobiae	*Verrucomicrobia*		Light/dark box test	Increased number of entries in the dark	
									p	*Verrucomicrobia*	*Verrucomicrobia*				
									s	*muciniphila*	*Verrucomicrobia*				
									g	*Akkermansia*	*Verrucomicrobia*				
									o	Turicibacteraies	*Firmicutes*				
									g	*Turicibacter*	*Firmicutes*				
									f	*Planococcaceae*	*Firmicutes*				
									g	*Anaerostipes*	*Firmicutes*	N/A			
								Decrease	g	*Bacteroides*	*Bacteroidetes*	LDA score			
									f	*Bacteroidaceae*	*Bacteroidetes*	(high to low)			
									f	*Lactobacilliaceae*	*Firmicutes*				
									g	*Lactobacillus*	*Firmicutes*				
									f	*Paraprevotellaceae*	*Bacteroidetes*				
									g	*Prevotella*	*Bacteroidetes*				
									f	*Clostridiaceae*	*Firmicutes*				
									g	*Ruminnococcus*	*Firmicutes*				
									s	*Ruminnococcus gnavus*	*Firmicutes*				
									f	*Hellicobacteraceae*	*Proteobacteria*				
									s	*Bacteroides acidifaciens*	*Bacteroidetes*	N/A			
									f	*Erysipelotrichaceae*	*Firmicutes*	N/A			
CSDS + SOC	3w	C57BL/6J	18w	Male	Harlan, UK	N/A	Cecal content	Increase	f	*Helicobacteracea*	*Proteobacteria*	N/A	Social interaction test	Decreased social interaction ratio	30066368
	SOC = 10 mice/cage, 24 h								g	*Prevotellaceae UCG 001*	*Bacteroidetes*	N/A	Three chamber test	No change	
	CSDS = 2 h/d								f	*Prevotellaceae*	*Bacteroidetes*	N/A	Open-field test	No change	
									o	Gastranaerophilales	*Melainabacteria*	N/A	Forced swimming test	No change	
								Decrease	g	*Ruminicoccaceae UCG 013*	*Firmicutes*	N/A	Female urine sniffing test	Decreased interaction time	
									g	*Intestinimonas*	*Firmicutes*	N/A	Marble burying test	No change	
													Elevated plus maze	No change	
													Tail suspension test	No change	
													Sucrose preference test	Increased sucrose preference	
													Novel object recognition test	No change	
													Hot plate test	No change	
RS	2 h/d, 5times/w, 6w	C57BL/6J	17-19w	Male	Harlan	N/A	Feces	Increase	f	*Lachnospiraceae*	*Firmicutes*	2.0900	Rotarod performance	No change	30579705
								Decrease	g	*Lactobacillus*	*Firmicutes*	2.9700			
RS	2 h/d, 7d	C57BL/6J	7-11w	Both	Jackson laboratory	Lab Diet 5053	Feces	Increase	f	*Bacteroidaceae*	*Bacteroidetes*	N/A	N/A	N/A	33196055
									o	Unclassical Burkholderiales	*Proteobacteria*	N/A			
								Decrease	f	*Ruminococcaceae*	*Firmicutes*	N/A			
									f	*Coriobacteriaceae*	*Actinobacteria*	N/A			
									f	*Unclassical Clostridiales*	*Firmicutes*	N/A			
RS	2 h/d, 10d	C57BL/6J	7-8w	Male	Orient Animal Breeding Center	N/A	Feces	Increase	p	*Proteobacteria*	*Proteobacteria*	N/A	Elevated plus maze	Decreased time spent in open arms	30224732
					(Seoul, Korea)				f	*Helicobacteraceae*	*Proteobacteria*	N/A	Light/dark box test	Decreased time spent in light area	
									f	*Enterobacteriaceae*	*Proteobacteria*	N/A	Marble-burying test	Increased percentage of marble buried	
									g	*Klebsiella*	*Proteobacteria*	N/A			
									g	*Helicobacter*	*Proteobacteria*	N/A			
								Decrease	p	*Actinobacteria*	*Actinobacteria*	N/A			
									p	*Bacteroidetes*	*Bacteroidetes*	N/A			
									f	*Lactobacillaceae*	*Firmicutes*	N/A			
RS	4–6 h/d, 21d	C57BL/6J	12-16w	Male	Australian Phenomics	N/A	Feces	Increase	f	*Lachnospiraceae*	*Firmicutes*	1.0030	Forced swimming test	Increased floating time	27090302
								Decrease	g	*Allobaculum*	*Firmicutes*	1.0840	Elevated plus maze	Decreased time into open arm	
									g	*Bifidobacterium*	*Actinobacteria*	1.0480			
									g	*Turicibacter*	*Firmicutes*	1.0350			
									g	*Clostridium*	*Firmicutes*	1.0070			
RS	16 h/d, 1d	C57BL/6N	6-8w	Male	Charles River Laboratories	N/A	Ileum	Increase	g	*Escherichia_Shigella*	*Proteobacteria*	LDA score	N/A	N/A	34795263
									f	*Enterobacteriaceae*	*Proteobacteria*	(high to low)			
									o	Enterobacteriales	*Proteobacteria*				
									p	*Proteobacteria*	*Proteobacteria*				
									c	Gammaproteobacteria	*Proteobacteria*				
									g	*Enterococcus*	*Firmicutes*				
									f	*Enterococcaceae*	*Firmicutes*				
									g	*Staphylococcus*	*Firmicutes*				
									g	*Streptococcus*	*Firmicutes*				
									f	*Streptococcaceae*	*Firmicutes*				
								Decrease	p	*Firmicutes*	*Firmicutes*	LDA score			
									c	Erysipelotrichia	*Firmicutes*	(high to low)			
									f	*Erysipelotrichaceae*	*Firmicutes*				
									o	Erysipelotrichales	*Firmicutes*				
									g	*lleibacterium*	*Firmicutes*				
									f	*Muribaculaceae*	*Bacteroidetes*				
									c	Bacteroidia	*Bacteroidetes*				
									o	Bacteroidales	*Bacteroidetes*				
									p	*Bacteroidetes*	*Bacteroidetes*				
									p	*Actinobacteria*	*Actinobacteria*				
							Cecum	Increase	f	*Enterobacteriaceae*	*Proteobacteria*	LDA score			
									o	Enterobacteriales	*Proteobacteria*	(high to low)			
									c	Gammaproteobacteria	*Proteobacteria*				
									p	*Proteobacteria*	*Proteobacteria*				
									g	*Mucispirillum*	*Deferribacteres*				
									p	*Deferribacteres*	*Deferribacteres*				
									f	*Deferribacteraceae*	*Deferribacteres*				
									o	Deferribacterales	*Deferribacteres*				
									g	*Escherichia-Shigella*	*Proteobacteria*				
								Decrease	c	Clostridia	*Firmicutes*	LDA score			
									o	Clostridiales	*Firmicutes*	(high to low)			
									f	*Lachnospiraceae*	*Firmicutes*				
									g	*Lachnospiraceae_NK4A136_group*	*Firmicutes*				
									f	*Muribaculaceae*	*Bacteroidetes*				
									f	*Erysipelotrichaceae*	*Firmicutes*				
									o	Erysipelotrichales	*Firmicutes*				
									c	Erysipelotrichia	*Firmicutes*				
									g	*lleibacterium*	*Firmicutes*				
									g	*Clostridiales_vadinBB60_group*	*Firmicutes*				
							Colon	Increase	g	*Bacteroides*	*Bacteroidetes*	LDA score			
									f	*Bacteroidaceae*	*Bacteroidetes*	(high to low)			
									f	*Enterobacteriaceae*	*Proteobacteria*				
									o	Enterobacteriales	*Proteobacteria*				
									g	*Escherichia_Shigella*	*Proteobacteria*				
									g	*Gammaproteobacteria*	*Proteobacteria*				
									p	*Proteobacteria*	*Proteobacteria*				
									f	*Deferribacteraceae*	*Deferribacteres*				
									p	*Deferribacteres*	*Deferribacteres*				
									o	Deferribacterales	*Deferribacteres*				
								Decrease	p	*Firmicutes*	*Firmicutes*	LDA score			
									o	Erysipelotrichales	*Firmicutes*	(high to low)			
									f	*Erysipelotrichaceae*	*Firmicutes*				
									c	Erysipelotrichia	*Firmicutes*				
									g	*lleibacterium*	*Firmicutes*				
									g	*Lachnospiraceae_NK4A136_group*	*Firmicutes*				
									f	*Lactobacillaceae*	*Firmicutes*				
									g	*Lactobacillus*	*Firmicutes*				
									o	Rhodospirillales/NA/NA	*Proteobacteria*				
									c	Alphaproteobacteria	*Proteobacteria*				
RS	3–4 h/d, 14d	C57BL/6J	13w	Male	Nanjing Medical University	N/A	Feces	Increase	g	*Akkermansia*	*Verrucomicrobia*	N/A	Forced swimming test	Increased immobility time	33535879
									g	*Anaerofustis*	*Firmicutes*	N/A	Sucrose preference test	Decreased sucrose preference	
								Decrease	g	*Parabacteroides*	*Bacteroidetes*	N/A	Elevated plus maze	Decreased duration in open arms	
									f	*Lachnospiraceae*	*Firmicutes*	N/A	Open-field test	Decreased center time	
									g	*Ruminococcus*	*Firmicutes*	N/A			
									f	*Unclassified_Ruminococcaceae*	*Firmicutes*	N/A			
WAS	1 h/d, 10 d	C57BL/6J	6-7w	Female	Charles River Laboratories	N/A	Feces	Increase	c	Gammaproteobacteria	*Proteobacteria*	3.0000	N/A	N/A	23470617
									p	*Firmicutes*	*Firmicutes*	2.0000			
								Decrease	p	*Bacteroidetes*	*Bacteroidetes*	2.0000			
							Small intestine	Increase	o	unclassified Clostridiales	*Firmicutes*	N/A			
							content		f	*Clostridiaceae*	*Firmicutes*	N/A			
									f	*Streptococcaceae*	*Firmicutes*	N/A			
								Decrease	f	*Lactobacillaceae*	*Firmicutes*	N/A			
									f	*Lachnospiraceae*	*Firmicutes*	N/A			
									p	*Unclassified Bacteroidetes*	*Bacteroidetes*	N/A			
									f	*Ruminococcaceae*	*Firmicutes*	N/A			
									p	*unclassified Firmicutes*	*Firmicutes*	N/A			
							Colon mucosa	Decrease	f	*Porphyromonadaceae*	*Bacteroidetes*	N/A			
									p	*unclassified Bacteroidetes*	*Bacteroidetes*	N/A			
							Colon content	Decrease	f	*Lactobacillaceae*	*Firmicutes*	N/A			
WAS	1 h/d, 10d	C57BL/6J	7–8w	Female	Charles River Laboratories	N/A	Feces	No change		No change	N/A	No change	Light/dark box test	No change	20966022
WAS	1 h/d, 1d	C57BL/6J	6–8w	Both	Jackson laboratory	N/A	N/A	N/A		N/A		N/A	Novel object recognition test	No change	31652348
													Light/dark box test	No change	
													Open-field test	Decreased the travel distance	

UCMS = unpredictable chronic mild stress, p = Phylum, CSDS = chronic social defeat stress, c = Class, RS = restraint stress, o = Order, WAS = water avoidance stress, f = Family, SOC: social overcrowding, g = genus, N/A: not applicable, s = species

Unpredictable chronic mild stress (UCMS) is an experimental condition that induces physiological and neurological changes that are similar to chronic and unresolved stress exposure. Mice generally display depressive-like behavior, similar to people with depression, with no apparent change in anxiety-like behavior [39–41]. Interestingly, the altered *Firmicutes* [39–41] and *Tenericutes* [40, 41] phyla are consistently observed in the UCMS animals. Of note, *Lactobacillaceae* seemed to be the main bacteria in *Firmicutes* that were decreased by UCMS [39, 41]. *Coprococcus* is a bacterial genus that was found to be reduced in UCMS mice [41] and the human depression cohort [42] (Table 1).

Chronic social defeat stress (CSDS) is a psychosocial stress with exceptional face, construct, and predictive validity. Behavioral outcomes after CSDS are complex, including an increase in depressive-like behavior, anxiety-like behavior, and a decrease in social behavior [43–46]. Likewise, the microbiome profiling shifted by CSDS was more complex than that shifted by UCMS. *Bacteroidetes* [44, 46] and *Helicobacteracea* [43–45] were increased after CSDS. In contrast, several bacteria in *Firmicutes*, such as *Ruminococcaceae* [44, 45], were altered after CSDS, except for *Lactobacillus* [43–46]. Social overcrossing (SOC) is a method that mimics increased housing density. The effect of SOC on behavior was minimal. Mice only showed increased speed in the elevated plus maze and entries to the dark chamber in the light/dark box [47]. However, the change in the microbiome after SOC was more dramatic. SOC increased the relative abundance of *Akkermansia muciniphila* and *Anaerostipes* genera and reduced the relative abundance of *Erysipelotrichaceae* family, *Lactobacillus*, and *Bacteroides acidifaciens* species [47]. The complex outcomes produced by social-related stressors could be due to the varied source of the intruders and the subtle difference in the experimental timelines (Table 1).

Restraint stress is a classical method of restricting rodent movement. Rodents develop anxiety- and depression-like behaviors after restraint stress [48–52]. While numerous bacterial taxa in the gut are altered, *Firmicutes* appears to be the most vulnerable bacteria that can be altered by chronic restraint stress, especially *Lactobacillaceae* and *Lachnospiraceae* family [48–51, 53, 54]. In addition, the *Proteobacteria* phylum was increased after chronic restraint stress [49, 53, 54]. Interestingly, restraint stress affected the microbiome differently, depending on the intestinal segment [54] (Table 1). WAS was a potent psychological stressor that disrupts gut epithelial tight junction integrity [55, 56]. The sole WAS did not produce much effect on the behavior compared to other stress models [56, 57]. However, the fecal microbiome was affected by WAS, with decreased *Bacteroidetes*, increased *Firmicutes*, and increased Proteobacteria. When analyzing the contents of the small intestine and colon, *Lactobacillaceae* and unclassified *Bacteroidetes* were lower in WAS mice [58].

Based on the studies we surveyed, the adaptation of the microbiome to stress could be influenced by different types of stress, duration of stress exposure, source of animals, diet, etc. (Table 1). Several bacterial taxa have been reported to have differences across studies after exposure to various types of stress. Stress exposure downregulates the relative abundance of *Porphyromonadaceae* [58, 59], *Lactobacillaceae* [39, 47, 49, 54, 58], *Ruminococcaceae* [44, 53, 58], and *Coriobacteriaceae* [43, 53] at the family level and *Parabacteroides* [51, 59] and *Lactobacillus* [40, 47, 48, 54] at the genus level. In contrast, stress exposure upregulated the relative abundance of *Streptococcaceae* [54, 58] and *Enterobacteriaceae* [49, 54] at the family level and *Anaerofustis* [40, 51] and *Helicobacter* [43, 49, 59] at the genus level. Among these studies, the *Lactobacillus* species was the most consistent bacterial taxa that was reduced in rodents following stress exposure.

### Levels of stress hormone in microbiome-depleted mice

Studies in mice have suggested that stress exposure alters the composition of the gut microbiome and shifts the bacterial taxa, which leads to another question: Do gut bacteria actively play a role in stress response regulation? To address this question, gnotobiotic and antibiotic-treated rodents are great models for “knocking out” the commensal microbiota constitutively or conditionally. Strikingly, most studies have suggested that the depletion of the microbiota in rodents enhances the stress response and increases the stress hormone corticosterone (Tables 2 and 3). Corticosterone is a glucocorticoid in rodents (cortisol in humans) that serves as a crucial steroid hormone secreted in response to stress [60].

**Table 2:** Corticosterone levels in germ-free (GF) rodents

Molecule	Species	Vendor	Strain	Sex	Diet	Age	Sample	Testing time	Treatment	Detection time	Changes	Reference (PMID)
Corticosterone	Mouse	Taconic Farms	C57BL/6N	Both	N/A	9–10 weeks	Serum	9:00–11:00	Baseline	N/A	No change	26218677
		Taconic Farms	C57BL/6N	Both	N/A	9–10 weeks	Serum	9:00–11:00	Maternal separation	N/A	Increase	26218677
		–	Swiss-Webster	Both	Sodium dodecyl sulphate diets 801,010	6–9 weeks	Plasma	N/A	Baseline	N/A	No change	22688187
		–	Swiss-Webster	Both	Sodium dodecyl sulphate diets 801,010	6–9 weeks	Plasma	N/A	Novel cage for 30 min	Immediately after stress	Increase	22688187
		Taconic Farms	Swiss/NIH	Both	N/A	6–8 weeks	Plasma	7:00–8:00	Baseline	N/A	Increase	32573321
		Taconic Farms	Swiss-Webster	Female	N/A	8 weeks	Plasma	N/A	48 h after arrival	N/A	Increase	21054680
		Taconic Farms	C57BL/6	Female	N/A	10–11 weeks	Plasma	8:00–14:00	Baseline	N/A	Increase	32391630
		Taconic Farms	C57BL/6	Female	N/A	10–11 weeks	Plasma	8:00–14:00	Restraint stress for 15 min	Immediately after stress, 45 min	No change	32391630
		CLEA Japan	BALB/c	Female	N/A	Adult	Plasma	N/A	Restraint stress for 1 h	Before, 1, 12 h	Increase (1 and 12 h)	11282153
		CLEA Japan	C57BL/6	Male	N/A	4–8 weeks	Feces	10:00–11:00	Baseline	4.5–8 weeks	Decrease	30680708
		Taconic Farms	C57BL/6N	Male	N/A	8 weeks	Plasma	N/A	Baseline	N/A	Increase	30675019
		CLEA Japan	IQI/Jic	Male	CMF	8–9 weeks	Serum	N/A	LPS	Before, 0.5, 1, 2, 3, 4, 12 h	Increase (0.5, 1, 2, 12 h)	10427685
		Jackson Laboratory	C57BL/6J	Male	5053 PicoLab Rodent Diet	11–15 weeks	Serum	13:00–17:00	Novel cage for 5 min	60 min	Increase	34194038
		Jackson Laboratory	C57BL/6J	Male	5053 PicoLab Rodent Diet	11–15 weeks	Serum	13:00–17:00	Novel cage + Social interactio for 5 min	60 min	Increase	34194038
		Taconic Farms	C57BL/6	Male	N/A	10–11 weeks	Plasma	8:00–14:00	Baseline	N/A	Increase	32391630
		Taconic Farms	C57BL/6	Male	N/A	10–11 weeks	Plasma	8:00–14:00	Restraint stress for 15 min	Immediately after stress, 45 min	Increase (immeidately after stress)	32391630
		CLEA Japan	BALB/c	Male	N/A	9 weeks	Plasma	N/A	Restraint stress for 1 h	Before,1, 1.5, 2, 2.5, 3 h	Increase (except before)	15133062
		CLEA Japan	BALB/c	Male	N/A	9 weeks	Plasma	N/A	Ether	Before, 0, 0.5, 1, 1.5, 2 h	No change	15133062
		Czech Academy of Sciences	BALB/c	Male	Altromin 1414	9 weeks	Plasma	9:00–13:00	Baseline	N/A	N/D	31798585
		Czech Academy of Sciences	BALB/c	Male	Altromin 1414	9 weeks	Plasma	9:00–13:00	Restraint stress for 2 h	Immediately after stress	Increase	31798585
		Czech Academy of Sciences	BALB/c	Male	Altromin 1414	9–13 weeks	Ex vivo intestine	13:00–17:00	anti-CD3	N/A	Decrease	33921780
	Rat	Anaxem	F344	Male	R03	11–13 weeks	Serum	N/A	Baseline	N/A	No change	24636517
		Anaxem	F344	Male	R03	11–13 weeks	Serum	10:00–16:00	Open-field test	Immediately after stress	Increase	24636517
		Charles River	F344	Male	NMF	8 and 40 weeks	Serum	9:30–11:30	Baseline and aging	N/A	Increase	7266076
		Zentralinstitut für Kunstgeschichte	F344	Female	N/A	130–180 g	Plasma	N/A	IL-1alpha	60 min	No change	8403498
		Zentralinstitut für Kunstgeschichte	F344	Female	N/A	140–180 g	Plasma	N/A	Adjuvant arthritis	18 days	Increase	8033416

N/A: not applicable

**Table 3:** The effect of antibiotics on corticosterone in rodents

Molecule	Species	Vendor	Strain	Sex	Diet	Age	Chemicals	Dosage	Route	Timing for antibiotic treatment	Sample	Testing time	Treatment	Detection	Changes	Reference (PMID)
Corticosterone	Mouse	Jackson Laboratory	C57BL/6J	Male	N/A	9 weeks	Bacitracin	0.5 mg/mL	Water	7–9 weeks old	Serum	Afternoon	Baseline	N/A	No change	27752130
							Neomycin	2 mg/mL								
							Vancomycin	0.2 mg/mL								
							Pimaricin	1.2 µg/mL								
		Samtaco Animal Breeding Center	C57BL/6	Male	N/A	6 weeks	Ampicillin	100 mg/kg	Gavage	Once a day for 2 days	Serum	N/A	EPM	120 min	Increase	29867078
		–	C57BL/6	Male	N/A	100 days	Ampicillin	1 mg/mL	Water	21–100 days old	Serum	N/A	Baseline	N/A	No change	34824332
							Vancomycin	5 mg/mL					Aβ1–42	N/A	No change	
							Neomycin	10 mg/mL								
							Metronidazole	10 mg/mL								
							Amphotericin B	0.1 mg/mL								
		Jackson Laboratory	C57BL/6	Male	N/A	23 weeks	Ampicillin	1%	Water	4–23 weeks old	Serum	N/A	Baseline (fasting for 5 h)	N/A	No change	34856844
							Neomycin	1%					Social overcrowding (fasting for 5 h)	N/A	Decrease	
		Taconic Farms	C57BL/6N	Male		8 weeks	Ampicillin	1 g/L	Water	6–8 weeks old	Serum	N/A	Novel cage for 3 h + Saline	90 min	No change	34401412
							Vancomycin	0.5 g/L					Novel cage for 3 h + Insuline	90 min	No change	
							Neomycin	0.5 g/L								
							Erythromycin	10 mg/L						N/A		
		Jackson Laboratory	C57BL/6J	Male	5053 PicoLab Rodent Diet	8–12 weeks	Ampicillin	1 g/L	Water	8–12 weeks old	Serum	13:00–17:00	Novel cage for 5 min	60 min	No change	34194038
							Vancomycin	0.5 g/L				13:00–17:00	Novel cage + Social interactio for 5 min	60 min	Increase	
							Neomycin	1 g/L								
							Metronidazole	0.5 g/L								
							Sucrose	1%								
		Charles River	C57BL/6J	Male	N/A	8–12 weeks	Ampicillin	1 g/L	Water	8–12 weeks old	Plasma	6:00	Baseline	N/A	Increase	23663780
							Vancomycin	0.5 g/L				18:00	Baseline	N/A	Increase	
							Neomycin	1 g/L								
							Metronidazole	1 g/L								
		Sichuan	BALB/c	Male	N/A	17–19 weeks	Ceftriaxone	250 mg/mL, 0.2 mL/d	Gavage	Once a day for 11 weeks	Serum	8:00–5:00	Gavage stress	60 min	Increase	32714875
		N/A	BALB/c	Male	N/A	70 days	Ampicillin	1 mg/mL	Water	21–28, 35–42, 49–56 days old	Serum	N/A	Baseline	N/A	No change	29872772
		N/A	BALB/c	Male	N/A	70 days	Cefoperazone	1 mg/mL	Water	21–28, 35–42, 49–56 days old	Serum	N/A	Baseline	N/A	No change	29872772
		N/A	BALB/c	Male	N/A	70 days	Ampicillin + Cefoperazone	1 mg/mL	Water	21–28, 35–42, 49–56 days old	Serum	N/A	Baseline	N/A	No change	29872772
		Japan SLC	ICR	Male	5% fat, 24% protein, and 54% carbohydrate	P12, 15, 21	Nebacitin [bacitracin-neomycin sulphate 2:1]	7 g/kg	Diet	ED14-21 days old	Plasma	N/A	Baseline	N/A	No change	34170061
		Japan SLC	ICR	Male	5% fat, 24% protein, and 54% carbohydrate	P39	Nebacitin [bacitracin-neomycin sulphate 2:1]	7 g/kg	Diet	ED14-39 days old	Plasma	N/A	Restraint stress for 20 min	0, 20, 60, 120 min	Decrease at 60 min	34170061
		Japan SLC	ICR	Pregnant female	N/A	Adult	Nebacitin [bacitracin-neomycin sulphate 2:1]	7 g/kg	Diet	ED14-21 days old	Plasma	N/A	Baseline	N/A	No change	34170061
		Sichuan	Kunming	N/A	N/A	21 days	Ampicillin	100 mg/kg	Gavage	Once a day (10–21 days old; 10–100 µL)	Serum	N/A	Baseline	N/A	Decrease	32775126
							Vancomycin	50 mg/kg								
							Neomycin	100 mg/kg								
							Bacitracin	100 mg/kg								
							Imipenem	50 mg/kg								
							Amphotericin B	1 mg/kg								
	Rat	Janvier SA	Wistar	Female	UAR pellets	200–225 g	Neomycin	0.50%	Water	12 days	Plasma	N/A	Baseline	N/A	No change	22541937
							Ampicillin	1%					Partial restraint stress for 2 h	Immediately	Decrease	
		CLEA Japan	ODS/Shi Jcl-od/od	Male	AIN93-rodent diet	8 weeks	Neomycin	1 mg/mL	Water	6–8 weeks old	Serum	N/A	Baseline	N/A	No change	32115449
							Vancomycin	0.5 mg/mL					Vitamine C	N/A	No change	
							Ampicillin	0.5 mg/mL								
		Shanghai Jiesijie	Sprague–Dawley	Male	N/A	200–250 g	Ampicillin	1 g/L	Water	28 days	Serum	N/A	Baseline	N/A	No change	32535221
							Vancomycin	500 mg/L					Intermittent electric shocks + noise for 14 days	Immediately	Decrease	
							Neomycin	1 g/L								
							Metronidazole	1 g/L								
		N/A	Sprague–Dawley	Male	Teklad Global 18% Protein Rodent diet,	23 weeks	Ampicillin	1 g/L	Water	10–16 weeks old	Serum	N/A	Forced swim test	0, 30, 45, 90 min	No change	27742460
					Product code 2018S		Vancomycin	500 mg/L								
							Ciprofloxacin HCl	20 mg/L								
							Imipenem	250 mg/L								
							Metronidazole	1 g/L								

N/A: not applicable

Germ-free (GF) mice, a model organism that was never exposed to bacteria in their lifetime, displayed elevated corticosterone levels after prolonged restraint stress exposure [61–63]. In addition, GF rodents exhibit elevated corticosterone levels under various stressful conditions, including maternal separation [64], environmental transition [65, 66], open-field test [67], social interaction [18], bacteria endotoxin LPS injection [68], and inducible-adjuvant arthritis [69].

However, not all studies have shown that GF rodents display excessive stress responses and higher corticosterone levels after exposure to stressful conditions [63, 70]. Consistent findings have not yet been obtained when it comes to measuring baseline corticosterone levels in GF rodents [63–65, 67, 70–74]. These studies discovered that the HPA axis is an influential mediator for gut microbes to alter host physiology; this raised the possibility of microorganisms in the gut playing a critical role in stress suppression (Table 2).

GF rodents clearly indicate that the depletion of gut microbiota leads to aberrant stress responses, including increased corticosterone, altered gene expression involved in stress signaling, and abnormal behavioral consequences. While GF models are valuable tools for studying microbial influences on stress-coping mechanisms, it is important to highlight the limitations of the GF model. GF animals, which lack exposure to microbes from birth, can exhibit several developmental differences compared to conventionally raised animals [75]. These distinctions include altered gut morphologies, an immature mucosal immune system, delayed oral tolerance development, deceleration of epithelial turnover, and neuroendocrine function alterations, especially during early life [75, 76]. The caveat regarding these differences recognizes the artificial nature of the GF model in the context of human physiology.

In parallel with GF mice, antibiotic administration was extensively adopted to clarify the role of gut microbiota in stress. Antibiotic administration is a powerful tool for controlling the timing of the elimination of commensal microbes [18, 20, 77, 78]. However, age, treatment time window, type, and dosage for antibiotic administration are critical factors for yielding consistent findings with GF rodents [76]. Furthermore, it is challenging to deplete gut microbes entirely because of the geographical preference of the GI tract for various species of gut bacteria [79]. Only a few studies were able to reproduce an enhanced stress response in GF mice using antibiotics (Table 3). Two studies adopted a similar antibiotic recipe with a broad-spectrum antibiotic cocktail (ABX), showing that chronic treatment of ABX in mice resulted in an increase in baseline corticosterone levels [80] and after social exposure [18]. Two other studies showed that an acute [81] or chronic [59] gavage dosing of a single antibiotic in mice increased corticosterone levels upon acute stress exposure. Intriguingly, developmental treatment of mice with antibiotics reduced corticosterone levels under various conditions [47, 82, 83]. The treatment of rats with antibiotics yielded a reduction in corticosterone, indicating a model-dependent effect [84, 85]. Other studies have shown that antibiotics do not affect corticosterone levels [86–90] (Table 3). While antibiotic administration is a potent approach for investigating the microbiome’s impact on brain and behavior, it demands careful consideration in experimental design.

### Dysregulation of stress response in the brain of microbiome-depleted mice

Dysregulation of the stress response in the brain has been widely observed in mice without commensal microbes. Several studies have investigated the gene expression levels of the glucocorticoid receptor (GR), CRH, and downstream signaling pathways in mice with gut microbial depletion. Crumeyrolle-Aria et al. showed that increased corticosterone levels and decreased GR mRNA levels in the CA1 hippocampus and dentate gyrus (DG) were observed in GF rats after exposure to stress [67]. Sudo et al. observed higher CRF expression in the hypothalamus of GF mice. GR gene expression was lower in the cortex, but not in the hypothalamus and hippocampus [62]. Luo et al. showed that hippocampal GR downstream signaling pathways, including *Slc22a5, Aqp1, Stat5a, Ampd3, Plekhf1, and Cyb561,* were upregulated in GF mice under baseline condition [91]. Gareau et al. showed that a reduction in neural activity in the hippocampal CA1 region was observed in GF mice when compared to SPF mice after WAS [57].

A recent finding illustrates that gut commensal microbes are required to restrain the host stress response and increase social behavior. The stress hormone corticosterone levels were elevated in GF mice after a short social interaction with a stranger mouse. Concurrently, the neural activity in several brain regions that are responsible for coping with stress was upregulated, including the PVN, hippocampal DG, and adrenodorsal BNST (adBNST) [18]. The upregulated stress hormones and neural activity were recapitulated in mice treated with ABX at the adult stage. Furthermore, this study showed that the immediate early genes were upregulated in the hippocampus (*Arc*, *Fos*, *cJun*, *JunB*, *Egr1, Egr2*, *Gadd45b*, *Gadd45g*, *Bdnf*) and hypothalamus (*Arc*, *Fos*, *Egr1*), but were downregulated in the brainstem (*cJun*, *JunB*, *Egr1*, *Gadd45b*, *Gadd45g*, *Bdnf) *of GF mice [18]. However, stress-related gene expression did not change in mice treated with antibiotics under baseline and stressful conditions [18]. Only C*rh* gene expression was upregulated in ABX mice after social encounters, whereas *Ucn* gene expression was upregulated in ABX mice after novel cage exposure [18].

**Fig. 2:**
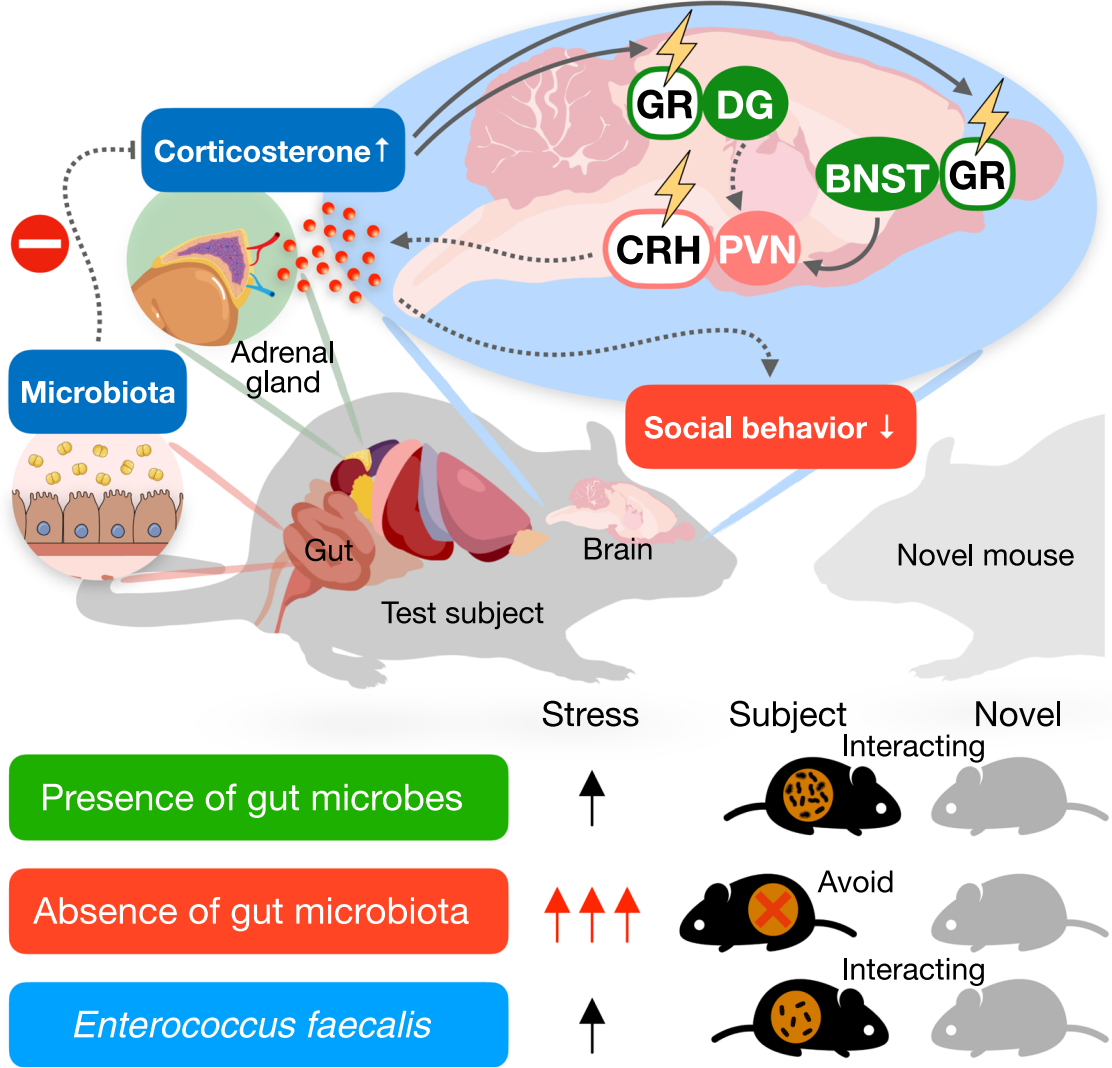
Gut commensal microbes are required to restrain the host stress response neurons increasing social behavior. Colonization of Enterococcus faecalis alleviated the social interaction-induced stress response and promoted the social behaviors toward the novel mouse. PVN: the paraventricular nucleus of the hypothalamus; BNST: the bed nucleus of the stria terminalis; DG: dentate gyrus; CRH: corticotropin-releasing hormone; GR: glucocorticoid receptor

To further investigate whether the interference of neurons in brain circuits can alter mouse stress hormones and social behavior, Wu et al. adopted a genetic ablation strategy and chemogenetic approach to disrupt the stress response neurons in ABX-treated mice. Genetic ablation of GR in the DG and adBNST restored social deficits and suppressed corticosterone levels in ABX mice (Fig. 2). In contrast, genetic ablation of GR in the hypothalamus decreases social behavior and increases corticosterone levels after social interaction [18]. Silencing the PVN CRH neurons in ABX mice suppressed the increase in corticosterone levels and prevented the development of social deficits (Fig. 2). These effects were not observed in adBNST CRH neurons from ABX mice [18]. Furthermore, adrenalectomy and pharmacological blockade of the GR and synthesis of corticosterone in microbial-depleted mice sufficiently restored their social interaction behavior [18]. Therefore, Wu et al. suggested that the dysregulation of social behavior and stress response in mice without a microbiome is more likely due to the altered neural activity in PVN CRH neurons, instead of alterations in stress-related gene expression or structural changes in PVN-associated neural circuits [18]. This study provides a defined pathway for stress coping by commensal microbes to drive host behavior (Fig. 2). Advances in neuroscience technologies have allowed scientists to precisely investigate the neural circuits regulated by microbiota and further discover the mechanisms involved in microbiome-mediated stress-associated neural circuits.

### Extra-adrenal steroidogenesis in the gut

While neurons in the brain in response to stressful conditions have been extensively explored, gut-derived stress signaling has not yet been fully elucidated. Glucocorticoids, a class of corticosteroids, are secreted mainly by the adrenal gland and partially by the extra-adrenal system [92, 93]. The amount of glucocorticoids released by the adrenal gland is far beyond the amount released by the extra-adrenal system. Although adrenal glucocorticoids play a role in the response to stress, the physiological role of extra-adrenal glucocorticoids in the intestine is still not understood.

The canonical steroidogenesis pathway for corticosterone in the adrenal gland involves a series of steps in the mitochondria. Cholesterol is converted to pregnenolone by two rate-limiting enzymes, steroidogenic acute regulatory protein (StAR) and cytochrome P450 family 11 subfamily A member 1 (CYP11A1). Pregnenolone is then catalyzed to progesterone and 11-deoxycorticosterone by 3β-Hydroxysteroid dehydrogenase (3β-HSD) and CYP21A2, respectively. Then, 11-deoxycorticosterone is catalyzed to corticosterone by CYP11B1 [94]. Corticosterone can also be produced by 11-dehydrocorticosterone with the enzyme 11β-hydroxysteroid dehydrogenase type 1 (11β-HSD1), and vice versa by 11β-HSD2 [95]. Brunner group demonstrated that the synthesis of extra-adrenal glucocorticoids is independent of the canonical adrenal glucocorticoid synthesis. First, the critical nuclear receptor and transcription factor steroidogenic factor-1 (SF-1) for adrenal steroidogenesis is absent in the intestine and is functionally replaced by liver receptor homolog-1 (LRH- 1) [96, 97]. Second, ACTH, the primary hormone secreted by the anterior pituitary gland; it stimulates steroidogenesis in the adrenal gland, but is not involved in intestinal glucocorticoid synthesis [98]. Based on the fundamental distinction in the biochemical process of synthesizing corticosterone, the physiological role of extra-adrenal glucocorticoids is considered to be independent of stress coping [92, 93, 99, 100].

Intestinal epithelial cells (IEC) are primary producers of extra-adrenal glucocorticoid synthesis [92, 93]. Strikingly, the small and large intestines and appendix express critical enzymes involved in steroidogenesis, including *Cyp11a1* and *Cyp11b1* [99]*.* Intestinal glucocorticoids are hypothesized to contribute to the intestinal microenvironment [93]. Brunner group showed that systemic immune challenges upregulate glucocorticoid synthesis and interact with the immune cells in the gut [99]. Anti-CD3 injection or viral infection upregulated steroidogenic enzymes *Cyp11a1*, *Hsd3b1*, *Cyp21*, *Cyp11b1*, and *Hsd11b1* and immuno-stimulated corticosterone production in the small intestinal mucosa [99]. Moreover, they found that pro-inflammatory cytokine tumor necrosis factor α (TNFα) and LPS-induced immune system activation promote steroidogenesis in the intestine [101, 102]. In contrast, Raddatz et al. showed that IL-1β was shown to inhibit glucocorticoid signaling in IEC in vitro models [103]. Treatment of IEC with dexamethasone, a GR agonist, increased its transepithelial electrical resistance without affecting the tight junction architecture. Increased barrier function due to glucocorticoid agonism could be compromised by co-treatment with cytokines [104]. However, chronic treatment with dexamethasone may interact with the culture time of IEC cell lines since it affects epithelial permeability and ultimately, alters the gene expression for the actomyosin cytoskeleton, tight junction, integrin, and cell cycle pathway [105]. Upon bacterial endotoxin LPS injection in mice prior to ex vivo culture, corticosterone levels produced by extra-adrenal tissues dramatically increased [100]. Therefore, the extra-adrenal glucocorticoids primarily have immunoregulatory functions as suggested by LPS injection studies, distinct from the participation in the canonical stress signaling.

Furthermore, in patients with inflammatory bowel disease (IBD), there is a notable reduction in the expression levels of 11β-HSD1 in the colon, suggesting that impaired intestinal glucocorticoid synthesis may contribute to IBD development [106]. Intestinal glucocorticoids also play a pivotal role in inhibiting tumor development and growth during the inflammatory phase. However, during the tumor phase, glucocorticoid synthesis mediated by *Cyp11b1* suppresses anti-tumor immune responses, promoting immune evasion. This presents a promising therapeutic target for tumor treatment [107]. These findings highlight the significant role of intestinal glucocorticoid synthesis in modulating gastrointestinal disorders.

Gnotobiotic rodents have provided clues as to how the loss of microbiota alters the stress response in the gut. Stress-associated gene expression in the intestine is altered in GF mice under baseline, immune challenge, and stress exposure conditions [70, 108, 109]. The expression of steroidogenesis genes in the pituitary gland, adrenal gland, and intestine was compared in SPF and GF mice under social defeat and acute restraint stress conditions. Briefly, the gene expression of *Crh* and *Ucn2* in the colon was upregulated in SPF mice, but unchanged in GF mice after social defeat stress, partially due to the baseline increase in GF mice. Interestingly, the downregulation of *Hsd11b1 *was observed in both SPF and GF mice after social defeat stress, regardless of increased baseline levels in GF mice [108]. Another study investigated the intestinal segment-specificity of steroidogenesis in the intestine of GF mice. Both acute restraint stress and the presence of microbiota alter *Nr5a2* (encoding LRH-1) and *Hsd3b2* expression in the ileum and colon. However, it appears that the genes for steroidogenesis are more robustly altered in the colon than in the ileum [70].

These studies suggest that intestinal steroidogenesis may be a crucial pathway by which the gut bacteria regulate stress responses. The precise mechanism by which bacteria in the GI tract affect the HPA axis remains unknown. Extra-adrenal steroidogenesis is a promising pathway for investigation.

### Circadian regulation of glucocorticoids and microbial impact

Circadian rhythms are intrinsic timekeeping systems governing a myriad of physiological processes, including the diurnal variations in glucocorticoid levels. These rhythms are not only influenced by endogenous factors but can also be significantly modulated by the gut microbiota. The levels of glucocorticoids fluctuate in accordance with the circadian rhythm in both physiological and pathological conditions. This pattern typically involves a peak in the early morning, followed by declining levels throughout the daytime. Several studies have reported on this circadian variation [110–115]. Moreover, clinical studies have suggested that patients with arthritis experience a state of hypercorticosterolism, as evidenced by elevated plasma cortisol levels measured in the morning compared to those measured at midnight [112, 115]. This observation appears to be synchronized with the presence of early morning stiffness in individuals with arthritis [112, 115]. Interestingly, Mukherji et al. characterized ileal IEC in corticosterone overproduction in ABX mice, revealing higher corticosterone levels at a time when ACTH was scarcely released [80]. Remarkably, corticosterone levels remained comparable in adrenalectomized ABX mice [18, 80]. This result indicated signal pathways involved in circadian clock regulation were disrupted in the ileal IEC of ABX mice, leading to hypercorticosterolism [80].

Circadian disruption driven by the microbiota has been observed in various disease conditions, including IBD and prediabetic syndromes [80, 116]. Microbiota can mediate the circadian disruption in mammals. Antibiotic treatment can ablate the microbiota, reprogramming the intestinal circadian transcriptome and rhythmic chromatin dynamic [117]. Another study demonstrated that the depletion of microbiota affected the crucial regulator of circadian rhythm, including a decrease of the transcripts of *Bmal1* and *Cry1*, and an increase the transcripts of *Per1* and *Per2*, while the transcript of *Clock* remained unaffected [80]. The disrupted signal pathways involved in circadian clock regulation resulted the hypercorticosterolism in ileal IEC [80]. This study indicated the deficiency of microbiota caused a prediabetic syndrome which was induced by ileal corticosterone overproduction and circadian disruption [80]. GF mice were observed of the lower level of circadian clock gene, such as *Bmal1*, *Clock*, *Per1*, and *Cry1* in the hypothalamus [118]. Exposure to bacterial metabolites may change circadian gene expression both in vitro and in vivo [118]. *Lactobacillus reuteri* alleviated the liver gene expression of *Nr1d1*, the core circadian gene encoding *REV-ERBα*, in the circadian dysrhythmia-induced polycystic ovary syndrome (PCOS) [119].

A constitutively active myosin light chain kinase (MLCK) in intestinal epithelia transgenic mice results in a colitis-prone phenotype, with an increased number of intraepithelial bacteria in the colonocytes of these mice [116]. Pai et al. reported that their microarray analysis revealed disruptions in the circadian rhythm in wildtype mice when they were co-housed with MLCK transgenic mice, in contrast to wildtype mice housed exclusively with other wildtype mice [116]. These disruptions were associated with changes in circadian gene expression in the colonic mucosa, including reduced *Nr1d1*, *Per1*, and *Per3*, in wildtype mice co-housed with MLCK transgenic mice [116]. Additionally, qPCR analysis demonstrated circadian gene expression with elevated *Arntl* and *Nfil3*, as well as reduced *Nr1d1*, in both colonic mucosa and purified colonocytes of wildtype mice co-housed with MLCK transgenic mice, compared to those exclusively housed with only wildtype mice [116]. The glucocorticoid enzyme *Cyp11a1* expression was decreased in the epithelial cell at specific time point in MLCK transgenic mice [116]. Furthermore, when invasive bacteria, found in increased numbers within the intraepithelial bacteria of MLCK transgenic mice, were co-cultured with Caco-2 cells, elevated levels of *Nr1d1* and *Nfil3* were observed [116]. This suggests that exposure to microbiota caused circadian disruption in the bacteria-epithelial co-culture system [116]. Taken together, this evidence suggests that the increased intraepithelial bacteria led to circadian disruption and glucocorticoid downregulation in the gut.

### Transmission of stress response from the gut to the brain via autonomic nervous system

In addition to the gut, the autonomic nervous system (ANS) is an essential pathway composed of sympathetic and parasympathetic nerves innervating the gut and brain, rapidly transmitting signals. ANS complements the body to maintain homeostasis and responds to various stimuli. The parasympathetic system is dominant for the "rest or digest" condition. This system is composed of specific cranial nerves, such as the optic nerve (III), facial nerve (VII), glossopharyngeal nerve (IX), vagus nerve (X), and pelvic splanchnic spinal nerve. Among the cranial and spinal nerves, the vagus nerve is the main component, with approximately 75% of the parasympathetic fibers in this system. Approximately 80% of afferent neurons and 20% of efferent neurons [120] in the vagus nerves innervate the GI tract. Moreover, the vagus nerves innervate the esophagus, lower airways, heart, aorta, liver, GI tract via the vagal branches [121]. The vagus nerve is the most rapid route for signal transduction among the pathways in gut-brain communication [122].

Leveraging advanced neurotechnologies, researchers can closely examine the fundamental roles of the ANS in healthy and disease states [122–124]. The parasympathetic vagus nerve is considered as the main interoceptive pathway in the GI tract [4]. The afferent vagus nerve ending is connected with the neuropod cells, which are responsible for enteroendocrine secretion and transduced luminal nutrient signaling in a millisecond fashion [122]. Besides nutrient sensing, GI stretch and gut motility are transmitted through vagal afferent neurons [125]. In addition to the primary function of the digestive system, the vagus nerve participates in other brain functions, including reward [123, 126], cognition [127], and satiety [128].

The causal relationship between the vagus nerve and the stress response has been demonstrated in several studies. Stimulation of the vagus nerve increases the serum corticosterone levels in rats [129, 130]. Genetically selective rat lines with altered glucocorticoid responsiveness display differential vagal tone following stress exposure [131]. In a human study, the injection of metyrapone, a drug that effectively blocks the critical enzyme to synthesize glucocorticoids in healthy subjects, dramatically reduced vagal-mediated heart rate variability [132]. The vagotomy procedure moderately altered nicotine-induced ACTH and corticosterone levels in a rat model [133]. The association between the vagus nerve and the stress response has been extensively investigated in immune challenge models. Subdiaphragmatic vagotomy effectively abolishes IL-1β-induced corticosterone elevation [134–136]. However, one report showed that vagotomy did not affect circulating cytokines and corticosterone when injected with LPS, suggesting a vagus-independent pathway [137]. Consistently, our study showed that subdiaphragmatic vagotomy cannot reverse ABX-induced social impairment or corticosterone levels [18]. Interestingly, a probiotic study found that ingestion of *Lactobacillus (L.) casei* strain Shirota was able to downregulate stress-induced glucocorticoids and relieve stress-associated symptoms in humans and rats. Moreover, treatment of *L. casei* strain Shirota in rats increased the vagal afferent nerve pulse in a dose-dependent manner and suppressed stress-induced CRF expression at PVN [138]. The differences between these findings can largely be attributed to different animal models, vagus nerve manipulations, and stimuli.

Strikingly, transcriptomic analysis by single-cell RNA sequencing revealed that the nodose and jugular ganglia expressed low levels of GR genes (*Nr3c1*) under baseline condition by single-cell RNA sequencing [139]. Interestingly, cell clusters with relatively high GR expression were functionally predicted to serve as GI tension sensors or mucosal chemo/mechano sensors [139]. However, GR expressing gastric vagal afferents, including the nodose ganglion and muscular/mucosal gastric vagal neurons, were found not to be affected by corticosterone in response to mechanical stimulation [140]. These data indicate that vagal afferent neurons express GR, but the functional role of glucocorticoid agonism in the GI tract remains unclear.

Sympathetic contributions to gut and gut microorganisms are not yet well-understood. One report showed that the depletion of the gut microbiota activated neural activity in the celiac-superior mesenteric ganglia (CG-SMG), the extrinsic sympathetic neurons responsible for GI tract innervation, thus altering gut motility [124]. Colonizing a specific community of bacteria, altered Schaedler flora, or *Clostridium spp.*, or administering gut fermentation metabolites short-chain fatty acids can suppress the activation of neurons in CG-SMG. Anatomically, vagal innervated brain regions are interconnected with brainstem nuclei critical for CG-SMG activation. Modulating vagal afferent signaling could alter gut sympathetic neural activity, revealing a complex neural innervation from the brain to the gut involving ANS [124].

In brief, the ascending and descending neural inputs of the parasympathetic and sympathetic nerves sense and respond to subtle changes in the lumen of the GI tract, including the commensal microbiota, in the modulation of higher brain functions beyond digestion. Taken together, these studies suggest that the stress-induced response of various compounds in the gut could potentially activate ANS and transmit signals to the brain.

### Neural pathways and neurotransmitters in gut-brain signaling via the vagus nerve

The neural pathways from the vagus nerve to the PVN CRH neurons are intricate [141]. The NTS serves as the primary relay for vagal afferent signals connecting to the forebrain [142]. Buller et al. showed that lesions within the NTS significantly decreased c-Fos expressions in PVN CRH neurons when exposed to systemic IL-1β [142]. Adrenergic and noradrenergic neurons were shown to bridge the connection between NTS and PVN. Chen et al. indicated that activation of noradrenergic neurons and adrenergic/neuropeptide Y neurons in NTS has been shown to modulate feeding behavior [143]. Moreover, a recent study showed that activation of NTS noradrenergic neurons resulted in reduced intake of both regular and high-fat diets, while also increasing PVN CRH c-Fos expression and elevating plasma corticosterone levels. This activation of the neural pathway from NTS NE neurons to PVN neurons also led to a decrease in chow food intake [144]. On the other hand, several studies have shown that preproglucagon neurons in NTS bridge the connection between NTS and CRH. Preproglucagon neurons are the primary source of glucagon-like peptide-1 (GLP-1) in the brain, a well-known gut hormone in the periphery [145]. Tracing studies confirm that preproglucagon neurons in NTS project to the PVN [146–149]. Reciprocally, the PVN contains a high density of GLP-1 receptors (GLP-1R), with colocalization observed in PVN CRH neurons [148]. To prove the functionality of this circuit, activation of NTS PPG neurons through chemogenetics or optogenetics directly stimulates PVN CRH neurons and suppresses food intake [150]. Furthermore, leptin-deficient mice exhibited increased NTS PPG neuron input to the PVN, resulting in higher c-Fos expression in PVN neurons [151]. In addition, intraperitoneal injection of the other gut hormone cholecystokinin (CCK) increased c-Fos expression in both NTS noradrenergic and PVN CRH neurons [152, 153]. The activity of PVN CRH neurons was increased during fasting conditions but was suppressed when the individual was in a fed state [13], suggesting that gut peptides may stimulate vagal terminals and alter forebrain neural activity. These findings collectively highlight the direct projections from NTS to the PVN CRH neurons.

Within the intricate framework of the gut-brain axis, a crucial aspect is the involvement of neural active molecules and their receptors in the gut that transmit signals to the brain. These molecules can be categorized into three main groups: neurotransmitters, gut peptides, and immune molecules. For neurotransmitters, serotonin (5-HT) within the gut primarily released by enterochromaffin cells [154]. It is tightly regulated by commensal microbiota [155] and has the capacity to directly activate the vagus nerve through the 5-HT_3_R receptor [156, 157]. Moreover, oral administration of selective serotonin reuptake inhibitors (SSRI) has been shown to increase the firing rate of vagal afferent neurons [158]. Notably, gastric distension has been observed to enhance c-Fos expression in the NTS and PVN. This effect can be mitigated through the intravenous injection of a 5-HT_3_R antagonist [159]. Additionally, intragastric administration of glutamate can activate gastric vagal afferent neurons, with the activation being notably hindered by pharmacological blocking of the 5-HT_3_R [160].

Gut peptides, including leptin, ghrelin, CCK, GLP-1, and peptide YY (PYY) are other well-known factors capable of activating the vagus nerve. Receptors for these gut peptides, such as the leptin receptor (LepR), GLP-1 receptor (GLP-1R), CCK receptor (CCKR), ghrelin receptor (GHSR), and Y2 receptor (Y2R), are expressed in nodose ganglion cells and the NTS region [125, 141, 161–166]. Ghrelin has been shown to decrease vagal afferent activity [161], while leptin, CCK, and GLP-1 were found to increase vagal afferent activity [77, 167–169]. Furthermore, vagal afferent neurons have the ability to function as chemosensors and mechanosensors to monitor changes within the gut lumen through gut peptide signaling [125, 141, 170]. Nutrients are also capable of activating vagal afferent neurons. For instance, nutrients like sucrose have been demonstrated to transmit signals through the sodium-dependent glucose cotransporter 1 (SGLT1) on CCK-labeled neuropod cells, subsequently activating the vagus nerve through glutamatergic neurotransmission [171, 172]. The mechanical stretching of the digestive tract, including the stomach and intestine, induces in vivo calcium activity in vagal ganglia neurons [125]. This study further identified that GLP-1R neurons primarily detect mechanical signalling, while GPR65 neurons primarily detect perfused nutrients and serotonin, which are then transferred to the NTS region [125].

For the immune molecules, the activation of vagal terminals in the gut has been notably associated with pro-inflammatory cytokines and bacterial endotoxin. For instance, intravenous injection of IL-1β resulted in a significant increase in c-Fos expression within the nodose ganglion, while concurrently elevating the discharge activity of gastric vagal afferent neurons, all mediated by a prostaglandin-dependent mechanism [173]. Similarly, intraportal administration of IL-1β was found to augment the discharge rate of the hepatic branch of vagal afferent nerves [174]. The specificity of vagal sensory neuron responses to IL-1β was further demonstrated by using IL-1R knockout mice, highlighting the pivotal role of the IL-1R receptor [175, 176]. Moreover, the action potential recording within the cervical vagus nerve was notably absent in TNF receptor knockout mice when exposed to TNF [175]. Toll-like receptor 4 (TLR4), known for mediating the signalling of bacterial endotoxin LPS [177, 178], is expressed in vagal afferent neurons [179, 180]. The administration of LPS promptly induced calcium influx in cultured vagal neurons [181], and notably, it heightened the release of calcitonin gene-related peptide (CGRP) in vagal afferent neurons through the TLR4 pathway [182].

In summary, the intricate neural pathways and neurotransmitters in the gut-brain connection via the vagus nerve have diverse roles. Neurotransmitters, gut peptides, nutrients, mechanosensation, and cytokines influence vagal activity through specialized receptors. This complex interplay shapes various physiological responses, impacting stress, appetite, and sensation. These mechanisms provide insights into the gut-brain axis, with implications for health and diseases.

### Probiotic- and bacteria-based effects for stress response

Microbiota-based supplements such as probiotics have been shown to alleviate stress responses by downregulating stress hormones. Interestingly, *Lactobacillus* bacteria are widely used as probiotics to alleviate stress responses, which are coincidentally observed to be downregulated when animals are exposed to stress (Table 1). Therefore, we summarized the current findings on using probiotics to alleviate stress responses and regulate the stress hormone corticosterone (Table 4).

**Table 4:** The effect of probiotics on corticosterone in rodents

Treated microbiota	Strain	Age	Sex	Routes	Period of probiotic treatment	Stress model	Stress exposure time	Stess hormone	Outcome	Reference (PMID)
*Bifidobacterium*	BALB/c mice	9 weeks	Male	Gavage	3 weeks (once per day)	Restraint stress	1 h	Decrease	Decrease ACTH and corticosterone	15133062
*Bifidobacterium adolescentis* IM38	ICR mice	7 week	Male	Gavage	3 days (once a day)	Immobilization	2 h	Decrease	Decrease corticosterone	28969445
*Bifidobacterium adolescentis* NK98	C57BL/6 mice	8 week	Male	Gavage	10 days (once a day)	Immobilization	10 days (2 h daily)	Decrease	Decrease corticosterone	30224732
*Bifidobacterium bifidum* G9-1 (BBG9-1)	Sprague–Dawley rats	P20	Both	Gavage	P4-P19 (once day)	Restraint stress	1 h	Decrease	Decrease corticosterone	34711869
*Bifidobacterium breve s*trains CCFM1025	C57BL/6J mice	12 week	Male	Gavage	5 weeks (once daily)	UCMS	5 weeks	Decrease	Decrease corticosterone	32258258
*Bifidobacterium breve *strains M2CF22M7	C57BL/6 mice	12 week	Male	Gavage	5 weeks (once daily)	UCMS	5 weeks	Decrease	Decrease corticosterone	30743155
*Bifidobacterium longum* NK46	C57BL/6 mice	6 week	Male	Gavage	5 days (once a day)	Immobilization	2 days (12 h daily)	Decrease	Decrease corticosterone	31564078
*Bifidobacterium longum* 1714	Human	25.5 year old	Male	Gavage	4 weeks	Socially evaluated cold pressor test	10 min	Decrease	Decreased stress hormone levels after stress-induced events	27801892
*Bifidobacterium pseudocatenulatum* CECT 7765	C57BL/6 mice	3 week	Both	Gavage	P2 to P21	Maternal seperation	20 days (3 h daily)	Decrease	Decrease corticosterone	28512033
*Enterococcus faecalis* 2001	ddY mice	N/A	Male	Gavage	14 days (once daily)	Dextran sulfate sodium	7 days	N/A	Alleviate colitis-induced enteric neurotransmission and pathologies	31672153
*Enterococcus faecalis* EC-12 strain	C57BL/6J mice	12 week	Male	Diet	4 weeks	N/A	N/A	N/A	Reduced anxiety like behavior and altered the receptors for norepinephrine and vasopressin in the prefrontal cortex	31931033
*Enterococcus faecalis*	C57BL/6J mice	11–15 (GF)	Male	Gavage	3 weeks	Social behavior	5 min	N/A	Decrease corticosterone (ABX), no change in GF mice	34194038
*Enterococcus faecalis*	C57BL/6J mice	12–16 (ABX)	Male	Gavage	3 weeks	Social behavior	5 min	Decrease	Decrease corticosterone (ABX), no change in GF mice	34194038
*Enterococcus faecalis* strains, K9 and CP-1	CF-1 mice	6–8 week	Female	I.P	Once	Inject peritoneally with pathogenic bacteria	N/A	Increase	Increased the corticosterone in an acute manner	16522776
*Enterococcus faecalis* SF3B strain	Wistar rats	8–11 weeks	Male	Diet	14 days	N/A	N/A	N/A	Alleviate colitis-induced enteric neurotransmission and pathologies	26550572
*Lactobacillus casei* 54–2-33	Sprague–Dawley rats	5 week	Male	Water	2 weeks	Elevated-plus maze	5 min	Decrease	Decrease corticosterone	28694176
*Lactobacillus casei* DKGF7	Wistar rats	12 week	Male	Gavage	4 weeks (Daily)	Chronic restraint	4 weeks (2 h daily)	Decrease	Decrease corticosterone	33572194
*Lactobacillus casei* strain Shirota	F344 rats	10–11 week	Male	Gavage	2 weeks (Daily)	WAS	1 h	Decrease	Decrease corticosterone	26896291
*Lactobacillus casei* strain Shirota	Human	22.8–23 year	Both	Milk	8 weeks	Academic stress	N/A	Decrease	Decrease cortisol	26896291
*Lactobacillus farciminis* ML-7	Wistar rats	N/A	Female	Gavage	2 weeks (once per day)	Partial restraint stress	2 h	Decrease	Suppress the activated HPA axis	22541937
*Lactobacillus fermentum* CECT5716	Sprague–Dawley rats	20–21 days	Both	Gavage	Postnatal 6 to postnatal 21 (once a day)	WAS	2 h	Decrease	Decrease corticosterone	28,370,715
*Lactobacillus helveticus* NS8	Sprague–Dawley rats	N/A	Male	Water	26 days	Chronic restraint	3 weeks (6 h daily)	Decrease	Decrease corticosterone	26408987
*Lactobacillus johnsonii* BS15	C57BL/6 mice	7 week	Male	Gavage	28 days (once per day)	Immobilization	28 days (1 h daily)	Decrease	Decrease corticosterone	34122081
*Lactobacillus johnsonii* isolates	C57BL/6 mice	5 week	Male	Gavage	5 days (once per day)	Immobilization	2 days (12 h daily)	Decrease	Decrease corticosterone	30979031
*Lactobacillus mucosae *NK41	C57BL/6 mice	6 week	Male	Gavage	5 days (once a day)	Immobilization	2 days (12 h daily)	Decrease	Decrease corticosterone	31564078
*Lactobacillus paracasei* Lpc-37	Swiss mice	10 week	Male	Gavage	5 weeks (Daily)	Chronic daily restraint	3 weeks	Decrease	Decrease corticosterone	31765723
*Lactobacillus paracasei* PS23	C57BL/6J mice	4 week	Both	Gavage	4 weeks	Early-life stress	2 weeks	Decrease	Decrease corticosterone	30882243
*Lactobacillus paracasei* DKGF1 with *Opuntia humifusa* extract	Wistar rats	12 weeks	Male	Oral	4 weeks	Chronic restraint stress	4 weeks (1 h daily)	Decrease	Decrease corticosterone	33092151
*Lactobacillus paracasei* HT6	Wistar rats	33 days	Both	Gavage	14 days (Daily)	Early-life stressful social experience	33 days	Decrease	Decrease corticosterone, ACTH in serum and GR expression in the brain	34531716
*Lactobacillus plantarum* PS128	C57BL/6J mice	8–12 week	Both	Gavage	4 weeks (Daily)	Early-life stress	13 days (3 h per day)	Decrease	Decrease corticosterone	26620542
*Lactobacillus reuteri* ATCC-PTA-6475	C57BL/6 and swiss mice	12 week	Both	Water	4 weeks	Wound healing	N/A	Decrease	Decrease corticosterone	27825953
*Lactobacillus reuteri* exopolysaccharide	C57BL/6 mice	6 week	Male	Gavage	5 days (once a day)	Ampicillin treatment	2 days (once a day)	Decrease	Decrease corticosterone	29867078
*Lactobacillus reuteri* NK33	C57BL/6 mice	8 week	Male	Gavage	10 days (once a day)	Immobilization	10 days (2 h daily)	Decrease	Decrease corticosterone	30224732
*Lactobacillus rhamnosus* GG	C57BL/6 mice	16 week	Male	Gavage	8 weeks (Daily)	High-fat diet	8 weeks	Decrease	Decrease corticosterone	34064242
*Lactobacillus rhamnosus* JB-1	BALB/c mice	10–12 week	Male	Water	28 days	Acute restraint	30 min	Decrease	Decrease corticosterone	34175326
*Lactobacillus rhamnosus* JB-1	BALB/c mice	10–12 week	Male	Water	28 days	Maternal separation	30 min	Decrease	Decrease corticosterone	29867313
*Lactobacillus rhamnosus* GG	Sprague–Dawley rats	12 week	Male	Water	3 weeks	Acute restraint	30 min	Decrease	Decrease corticosterone	33343931
*Escherichia coli*	Swiss/NIH mice	6–8 week	Both	Gavage	Monocolonized	N/A	N/A	Decrease	Reduced the basal levels of corticosterone	32573321
*Escherichia coli*	C57BL/6 mice	6 week	Male	Gavage	5 days (once daily)	N/A	N/A	Increase	Increased the baseline corticosterone levels	30224732
*Klebsiella oxytoca*	C57BL/6 mice	6 week	Male	Gavage	5 days (once daily)	N/A	N/A	Increase	Increased the baseline corticosterone levels	29867078
*Bifidobacterium animalis* subsp *actis* BB-12®48 with *Propionibacterium jensenii* 702	Wistar rats	24 days	Both	Water	10 days (Dam)	Maternal separation	PND 2–14 (3 h daily)	Increase	Increased the corticosterone in female, not male	23071537
*Bifidobacterium longum R0175 (Probio'Stick®) and Lactobacillus helveticus* R0052	C57BL/6	8–10 week	Male	Gavage	2 weeks (once daily)	WAS	4 days (1 h daily)	Decrease	Decrease corticosterone	24372793
*Bifidobacterium longum, Lactobacillus helveticus, Lactobacillus rhamnosus, Lactobacillus casei*	Sprague–Dawley rats	10–12 week	Male	Gavage	4 weeks (once daily)	UCMS	28 days	Decrease	Decrease ACTH and corticosterone	33913925
*Lactobacillus delbrueckii and Lactobacillus fermentum (Heat-killed)*	C57BL/6	8 week	Male	Diet	9 weeks	Forced swim test	4 min	Decrease	Decrease corticosterone	30597248
*Lactobacillus plantarum* CCFM8610 and *Lactobacillus casei* M2-01-R02-S01 (M2S01)	C57BL/6	N/A	Male	Gavage	4 weeks (Daily)	WAS and gavage of *Citrobacter rodentium*	2 weeks (1 h daily)	Decrease	Decrease corticosterone	33427835
*Lactobacillus plantarum* LRCC5310*, Lactobacillus plantarum* LRCC5314*, and Lactobacillus gasseri* BNR17	C57BL/6	20 week	Male	Gavage	Twice daily for 12 weeks	Chronic cold stress and high-fat diet	12 weeks	Decrease	Decrease corticosterone	34001561
*Lactobacillus rhamnosus* strain R0011 (95%) and *Lactobacillus helveticus* strain R0052 (5%)	Sprague–Dawley rats	60–70 days	Both	Oral/Rectal	16 days (twice a day)	Acute water avoidance	30 min	Decrease	Decrease corticosterone	17339238

GF = germ-free, ABX = antibiotic cocktail, UCMS = unpredictable chronic mild stress, WAS = water avoidance stress, I.P. = intraperitoneal injection, N/A: Not applicable

*L. rhamnosus* has been used as a probiotic for several decades. *L. rhamnosus GG* alleviated acute restraint stress-induced corticosterone in maternal separation rats [183] and high-fat diet mice [184]. *L. rhamnosus* JB-1 reduced acute restraint stress-induced corticosterone levels through the subdiaphragmatic vagus nerve [185] in a strain-dependent manner [186]. The rat pups showed high corticosterone levels immediately after maternal separation. The increase in corticosterone levels can be prevented by oral administration of *L. rhamnosus* *strain R0011* (95%) and *L. helveticus* *strain R0052* (5%) (Lacidofil®) [187].

In addition to the *rhamnosus species, paracasei, plantarum, casei, *and other species have been shown to modulate stress in various models. Administration of *L. paracasei Lpc-37* [188] chronically decreased corticosterone levels induced by chronic daily restraint stress. *L. paracasei HT6* effectively prevented early life stressful social experience-induced changes in brain GR expression [189]. *L. paracasei* *PS23* [190] and *L. plantarum PS128* [191] reduced corticosterone levels induced by early life stress. *L. casei* *strain Shirota* reduced WAS-induced corticosterone levels in rats and academic stress-induced cortisol levels in humans [138]. *L. casei* *DKGF7* suppresses chronic restraint stress-induced corticosterone [192]. *L. plantarum CCFM8610* and *L. casei* *M2-01-R02-S01 (M2S01)* suppressed corticosterone levels in irritable bowel syndrome (IBS) models induced by WAS and *Citrobacter rodentium* [193]. *L. plantarum* *LRCC5310*, *L. plantarum LRCC5314*, and *L. gasseri BNR17* suppressed the elevation of corticosterone induced by chronic cold stress and high-fat diet [194]. *L. reuteri* exopolysaccharide suppresses ampicillin-induced corticosterone [81]. *L. reuteri* *ATCC-PTA-6475* downregulated corticosterone levels during wound healing [195]. *L. reuteri NK33,* *L. johnsonii* isolates*, L. johnsonii* *BS15*, and *L. mucosae* *NK41* suppressed corticosterone elevation induced by immobilization stress [49, 196–198]. *L. fermentum CECT 5716* alleviated the corticosterone levels induced by WAS and maternal separation [199]. Treatment with heat-killed *L. fermentum* and *L. delbrueckii* (*ADR-159*) decreased the baseline levels of corticosterone and increased sociability [200]. *L. helveticus* *NS8* reduces chronic restraint stress-induced corticosterone [201]. Treatment with *L. farciminis* *ML-7* successfully suppressed the activation of the HPA axis induced by partial restraint stress [84]. However, not every *Lactobacillus* species produces a downregulating effect on the stress response, including *L. paracasei N1115* [83], *L. plantarum* *LP12407* [188], *L. plantarum* *LP12418* [188], *L. salivarius* *UCC118* [202], *L. casei* *CRL431* [203], *L. salivarius* *HA113* [204]. Moreover, the renowned probiotic *L. rhamnosus* JB-1 was not able to change cortisol and release stress compared with the placebo group in humans [205].

In addition to *Lactobacillus* species*, Bifidobacterium (B.)* is another genus of bacteria that has been extensively investigated for stress regulation. Monocolonization of *B.* subtilis in GF mice attenuated the increase in restraint stress-induced ACTH and corticosterone levels [62]. *Bifidobacterium adolescentis NK98,* *B. adolescentis IM38*, and *B. longum* *NK46* suppress corticosterone elevation induced by immobilization stress [196, 198, 206]. *B. pseudocatenulatum* *CECT 7765* [207] and *B. bifidum* *G9-1 (BBG9-1)* [208] alleviated maternal separation-induced elevation in corticosterone levels. *B*. *breve strains M2CF22M7* [209] and *CCFM1025* [210] reduced the UCMS-induced corticosterone production. In a clinical study, the administration of *B. longum 1714* decreased stress hormone levels after stress-induced events [211]. Similarly, not all *Bifidobacterium* species are involved in stress regulation, including *B. infantis* *35624* [202, 212, 213], *B. breve* *UCC2003* [202], *B. longum* *1714* [214, 215], *B. breve 1205* [214, 215].

Probiotic mixtures that combine *Lactobacillus* and *Bifidobacterium species* also exert stress modulation effects. *L. helveticus* *R0052* and *B. longum* *R0175* (Probio'Stick®) reduced the elevation of corticosterone induced by WAS [204]. Treatment with probiotics combining *L. helveticu*s, *L. rhamnosus*, *L. casei*, *B. longum* suppressed ACTH and corticosterone levels in UCMS rats [216]. However, the mechanisms by which different bacteria interact with one another can be complicated. In contrast, maternal *B. animalis subsp. actis* *BB-12*^®^ with *Propionibacterium jensenii* *702* increased neonatal corticosterone [217].

Other bacteria, not commonly used as probiotics, have also been shown to modulate stress-induced hormones to a lesser extent. Monocolonization by *E. coli,* but not *Bacteroides fragilis* in GF mice reduced the basal levels of corticosterone [71]. Administration of *Klebsiella oxytoca* [81] and *E. coli* [49] increased baseline corticosterone levels. Wu et al. treated mice with a combination of antibiotics (ampicillin, vancomycin, and metronidazole; AVM) and found that the social behavior was preserved, and the stress response was restrained compared to mice treated with the full spectrum of ABX. The preserved social behavior and reduced stress response were transferred when transplanting the AVM gut microbiota to GF recipient mice, indicating that the gut bacteria in the AVM microbiome played an active role. *Enterococcus (E.) faecalis* was identified as the key bacterium that promotes social behavior and suppresses increased corticosterone levels during social encounters. Colonization of *E. faecalis* in ABX and GF mice can promote their social behavior, but only suppresses corticosterone levels in ABX, and not GF mice [18].

*E. faecalis *is a lactic acid bacterium that is resistant to antibiotics and many other stressors. The functional roles of *E. faecalis* in the host are multifaceted and strain-specific. *E. faecalis* is a well-known pathogen commonly found in urinary tract infections [218]. In contrast, *E. faecalis* has been widely used as a probiotic or food additive [219]. Interestingly, several studies have shown that *E. faecalis* can modulate the nervous system and host behavior. *E. faecalis EC-12* strain reduces the anxiety response and alters the receptors for norepinephrine and vasopressin in the prefrontal cortex [220]. *E. faecalis SF3B* strain [221] and *EF-2001* [222] strains have been shown to alleviate colitis-induced enteric neurotransmission and pathologies. In addition, *E. faecalis* can synthesize tyramine and-phenylethylamine, two neuroactive molecules known as trace amines and are considered to be able to modulate the host nervous system [223–226]. Substance P stimulates the production of tyramine and lactic acid in *E. faecalis V583* strain and enhances cytotoxicity and bacterial translocation in an intestinal in vitro model [227]. *E. faecalis AG5* can increase both long- and short-chain fatty acids in the host, which might indirectly affect the nervous system through an indirect fashion [228]. One report found that infection of mice with pathogenic *E. faecalis* strains, *K9* and *CP-1*, increased corticosterone in an acute manner, suggesting that *E. faecalis* can alter glucocorticoid signaling in the host [229]. Clinically, *E. faecalis* was present in 89.3% of healthy controls, whereas only in 58.3% of neurodevelopmental disorders, 58.3% of mixed specific developmental disorders, and 55.6% of expressive and receptive language disorder [230]. In addition, the administration of *E. faecalis* did not produce any effect on repetitive behavior and anxiety-like behavior in the offspring of maternal immune activation [231].

Altogether, the molecular and cellular mechanisms by which gut bacteria exert their effects on host emotion and stress responses will be investigated in the future. Despite the remarkable effects of microbiota on the HPA axis in animal studies, more clinical studies are required to support the concept of using probiotics to alleviate stress levels in humans.

### Prebiotic- and synbiotic-based effects for stress response

Prebiotics are non-digestible ingredients derived from food that have been used to promote the growth of microbes, mostly in the GI tract. Synbiotic treatment combines prebiotic and probiotic treatments to synergistically affect the host. Previous studies have shown that both prebiotic and synbiotic treatments can alter the corticosterone levels in rodent models. Few studies have investigated the interactions between prebiotics and stress exposure and their implications in the control of corticosterone levels.

Burokas et al. demonstrated that treatment with fructo-oligosaccharides (FOS) and galacto-oligosaccharides (GOS) produces anxiolytic and antidepressant effects in adult mice. Moreover, acute stress-induced corticosterone by forced swim test was effectively downregulated by GOS and the combination of FOS + GOS [232]. Interestingly, the relative abundances of *Akkermansia*, *Bacteroides,* and *Parabacteroides* were increased in the FOS and GOS treatments, while the relative abundances of *Desulfovibrio*, *Ruminococcus*, *Allobaculum*, *Turicibacter*, *Lactobacillus*, and *Bifidobacterium* were decreased by FOS + GOS [232]. However, two other studies using different compounds of prebiotics did not yield an inhibitory effect on corticosterone induced by inescapable stress (GOS, polydextrose, and the glycoprotein lactoferrin) [233] or by social disruption stress (human milk oligosaccharides 3’ sialyllactose or 6’ sialyllactose) [234]. We speculate that various compounds, treatment duration, and onset of treatment can influence the effects of prebiotics.

In addition to stress exposure, Liu et al. showed that chronic treatment with mannan oligosaccharide (MOS) decreased the baseline levels of corticosterone and CRH in the serum of a 5xFAD transgenic Alzheimer’s disease mouse model but not in wild-type mice. Furthermore, they found that butyrate levels in the serum and feces were increased by MOS and negatively correlated with serum corticosterone [235]. However, another study by Rodrigues et al. showed that MOS treatment decreased plasma corticosterone levels in wild-type Whistar rats during adulthood [236]. Interestingly, a drug-induced constipation rat model showed higher ACTH and lower corticosterone levels, which can be normalized by inulin and isomalto-oligosaccharide [237].

Synbiotic treatments with both prebiotics and probiotics are complex and have various combinations. To date, no study has used the same recipe with bacterial strains and prebiotic compounds for stress regulation. In a chronic stress model, Seong et al. found that combining maltodextrin *L. paracasei* *DKGF1* with *Opuntia humifusa* extract suppressed corticosterone levels induced by restraint stress in a time-dependent manner in rats exposed to chronic daily restraint stress [238]. Joung et al. found that the probiotic *L. gasseri 505* suppressed UCMS-induced corticosterone. Adding leaf extract *Cudrania tricuspidata* did not produce an additional effect on corticosterone [239]. In acute stress, Barrera-Bugueno et al. showed that co-treatment with *L. casei* *54-2-33* and inulin in rats decreased the elevated plus maze-induced corticosterone [240]. Few studies have adopted synbiotic strategies to alleviate the stress response and corticosterone, possibly due to the lack of a foundation regarding the mechanistic points of view on both probiotics and prebiotics.

### Direct modulation of biosynthesis and metabolism of steroids by microbiota

Steroidogenesis is a biosynthetic process that converts cholesterol to steroids in the host. In glucocorticoids, cholesterol is converted to corticosterone via several steps by several critical enzymes, including pregnenolone, progesterone, and deoxy-corticosterone. Corticosterone is then metabolized to aldosterone. Interestingly, several studies support the hypothesis that indigenous microbes directly modulate steroid synthesis in the host [241, 242]. This section discusses the potential bacterial candidates by which de novo bacteria convert cholesterol into steroids, which could interfere with the synthesis of glucocorticoid steroids.

The biosynthesis of steroids in bacteria is one way to directly influence steroid hormone levels. Pernigoni et al. found treatment with pregnenolone in the culture of *Ruminococcus (R.) gnavus*, *Bacteroides (B.) acidifaciens*, and *Clostridium (C.) scindens* under anaerobic conditions for 48 h can synthesize androgenic steroids; they measured the levels of steroid pathway intermediates using liquid chromatography-tandem mass spectrometry [242]. They detected hydroxypregnenolone, progesterone, dehydroepiandrosterone, and testosterone in bacterial conditioned media. Similarly, the same bacterial strain can metabolize hydroxypregnenolone to progesterone, dehydroepiandrosterone, and testosterone in vitro. However, *R. gnavus* and *B. acidifaciens* did not show any metabolic capability for cholesterol, cortisol, or aldosterone. Moreover, treatment with pregnenolone and hydroxypregnenolone in other commensal bacterial strains, including *E. faecalis*, *Enterobacter cloacae*, *Klebsiella pneumoniae 27*, *Proteus mirabilis*, *Serratia marcescens*, *Staphylococcus haemoliticus*, *E. coli*, yielded negative results, indicating the specificity of bacteria in the metabolism of steroid intermediates [242].

On the other hand, metabolizing steroid hormone can be the other pathway for bacteria to impact the levels of hormones in the host. Schaaf and Dettner isolated two *Bacillus* strains (HA-V6-3 and HA-V6-11) from the gut of a water beetle and showed that they were capable of metabolizing pregnenolone [243]. The other evidence demonstrated by Mosa et al. showing that indole and skatole, the two gut bacteria-derived metabolites of tryptophan fermentation, can inhibit CYP11A1, the rate-limiting enzyme for the steroidogenesis, to decrease pregnenolone [244]. Moreover, testosterone deficiency has been associated with depressive symptoms. Li et al. recently found that *Mycobacterium neoaurum* isolated from patients with depression can degrade testosterone into androstenedione [241]. A gene encoding 3β-hydroxysteroid dehydrogenase was identified in *Mycobacterium neoaurum* that degrades testosterone. 3β-hydroxysteroid dehydrogenase was introduced into *E. coli* to generate 3β-hydroxysteroid dehydrogenase-producing bacteria. Colonization of 3β-hydroxysteroid dehydrogenase-producing *E. coli* in ABX mice induced depressive-like behaviors [241]. A recent study done by Hsiao et al. investigated the effects of administering *Thauera sp.* strain GDN1, a betaproteobacterium with the ability to catabolize testosterone, to C57BL/6 mice. The results showed that the administration of strain GDN1 led to a significant reduction in serum androgen levels, as well as the detection of androgenic ring-cleaved metabolites in fecal extracts, suggesting that gut bacteria capable of androgen catabolism may regulate host circulating androgen levels and could potentially be utilized as probiotics in the alternative therapy of hyperandrogenism [245].

Although no study has shown that the specific bacteria's capability could directly influence corticosterone levels, the Hylemon laboratory at Virginia Commonwealth University discovered that *C. scindens*, a bacterium isolated from human feces, can convert glucocorticoids cortisol into androgens by a mechanism called side-chain cleavage [246]. A cortisol-inducible operon *desABCD* was identified in *C. scindens* ATCC 35704 using RNA-seq. *C. scindens* transports cortisol into bacteria via a sodium-dependent cortisol transporter encoded by *desD*. Cortisol can then be metabolized to 11β-hydro-xyandrost-4-ene-3,17-dione (11beta-OHA) by steroid-17,20-demolase, a putative transketolase encoded by *desAB*. 11beta-OHA can then be pumped out of the cell by ABC transporter [247]. It is not known whether there are bacteria that share a similar mechanism for converting corticosterone into other steroids. Another study from the Hylemon laboratory identified an enzyme corticosteroid 21-hydroxylase in the cell extracts of *Eggerthella lenta* (previously known as *Eubacterium lentum*). Interestingly, enzyme 21-hydroxylase uses deoxycorticosterone, deoxycortisol, dehydrocorticosterone, and corticosterone as substrates. This could be another mechanism by which microbes convert steroids to corticosterone in mammals. However, both *C. scindens* and *Eggerthella lenta* were neither reported in rodents after stress exposure (Table 1), nor were they found to colonize the guts of microbiome-deficient rodents.

From the perspective of biosynthesis and metabolism of corticosterone, some bacteria can promote corticosterone precursors, whereas others can have the opposite effect. Therefore, it remains challenging to identify a single pathway to clarify the hypercorticosterone found in GF and ABX-treated mice. The field faces a highly complicated situation in the gut that modulates stress hormones and stress-induced behavioral abnormalities.

### Clinical implication

Stress-related disorders, exemplified by irritable bowel syndrome (IBS), often involve microbial dysbiosis. IBS, a GI complication characterized by symptoms like abdominal discomfort, altered stool patterns, and accompanying anxiety, affects 5–10% of the population [248]. Despite extensive investigation, the precise etiology of IBS remains elusive, with recognized risk factors encompassing genetics, diet, psychological stress, and gut microbiome composition [249].

Studies reveal reduced α-diversity and notable differences in 21 bacterial species relative abundance in IBS patients compared to healthy controls [250]. IBS subtypes exhibited distinct alterations in gut microbiota-derived metabolites; constipation-predominant IBS (IBS-C) features reduced fecal bile acid concentration [251], whereas diarrhea-predominant IBS (IBS-D) showed elevated primary bile acids [252], which could be attributed to changes in the composition of the gut microbiota. Zhai et al. has shown that specific bacteria in IBS-D, like *Ruminococcus gnavus*, can stimulate serotonin biosynthesis by producing phenethylamine and tryptamine, accelerating gut motility [253]. Bercik group showed that *Klebsiella aerogenes*, found in some IBS patients, enhances histamine production, leading to visceral hyperalgesia through histamine 4 receptor signaling [254]. Notably, successful animal models for IBS can be established via fecal microbiota transplantation (FMT) from human IBS patient donors to GF recipients. This approach effectively replicates GI and anxiety symptoms observed in human IBS patients [254, 255].

Stress exposure is a known risk factor for the occurrence of IBS, commonly affecting gut motility and HPA axis [256]. IBS patients differ from healthy individuals in stress response hormone levels. Posserud et al. demonstrated acute mental stress leads to significant increases in plasma CRH and ACTH in IBS patients [257]. Further, Dinan group showed that ACTH and cortisol release augment in IBS patients following CRH infusion [258]. Colorectal distention (CRD), a method to detect visceral sensitivity [254], in animal model for IBS results in elevated c-Fos expression in PVN CRH neurons and increased plasma CRH, ACTH, and corticosterone levels [259]. Collectively, these findings indicate that individuals with IBS exhibit heightened stress hormone secretion and microbial dysbiosis compared to healthy subjects.

## Conclusion

Stress coping is an essential strategy for animals to face life-threatening events that may be harmful to their bodies. Stress dysregulation is strongly associated with affective diseases [3]. The COVID-19 pandemic has drastically escalated the global prevalence of stress-associated disorders and this impacts society profoundly [260]. Recent studies have suggested that the gut microbiota do not only arise in the background of stress exposure, but they also act as an “active modifier,” regulating the nervous and endocrine systems. We suggest that the fluttery feeling perceived as having “butterflies in the stomach” originates from the gut microbes. Gut microbes directly and locally modulate steroidogenesis, potentially altering stress hormone levels. Stress hormone signaling can then be propagated to the brain through defined pathways, extra-adrenal steroidogenesis, the autonomic system, and various bacterial components. Ultimately, the brain receives a message from the microbes and responds adequately to the PVN and other brain regions. Furthermore, the coping and adapting mechanisms determined by the brain can alter outputs based on behavior and endocrine function. Microbes can then be further adapted to the host physiology under stress. This controlling loop pathway, starting from the gut microbiota, is based on the current understanding of the interplay between intestinal microbes and stress. The molecular and cellular mechanisms, pathways, and circuits by which gut microbes regulate behavior remain largely unexplored. Identifying the key bacteria and bacteria-associated factors that contribute to and affect the stress response will benefit the innovation of alternative medicine using microbiome-based therapeutics.

## Data Availability

Not applicable.

## Abbreviations

ABX: Antibiotic cocktail
ACTH: Adrenocorticotropic hormone
adBNST: Adrenodorsal bed nucleus of the stria terminalis
ANS: Autonomic nervous system
AVM: Ampicillin, vancomycin, and metronidazole
BLA: Basolateral amygdala
CeA: Central nucleus of the amygdala
CG-SMG: Celiac-superior mesenteric ganglia
CRH: Corticotrophin-releasing hormone
CSDS: Chronic social defeat stress
FOS: Fructo-oligosaccharides
GF: Germ-free
GI: Gastrointestinal
GOS: Galacto-oligosaccharides
GR: Glucocorticoid receptor
HPA: Hypothalamic–pituitary–adrenal
IBS: Irritable bowel syndrome
IEC: Intestinal epithelial cells
IL: Interleukin
LPS: Lipopolysaccharide
MeA: Medial amygdala
MOS: Mannan oligosaccharide
mSTN: Medial subthalamic nucleus
PVN: Paraventricular nucleus of the hypothalamus
SOC: Social overcrossing
UCMS: Unpredictable chronic mild stress
WAS: Water avoidance stress
